# The eNanoMapper database for nanomaterial safety information

**DOI:** 10.3762/bjnano.6.165

**Published:** 2015-07-27

**Authors:** Nina Jeliazkova, Charalampos Chomenidis, Philip Doganis, Bengt Fadeel, Roland Grafström, Barry Hardy, Janna Hastings, Markus Hegi, Vedrin Jeliazkov, Nikolay Kochev, Pekka Kohonen, Cristian R Munteanu, Haralambos Sarimveis, Bart Smeets, Pantelis Sopasakis, Georgia Tsiliki, David Vorgrimmler, Egon Willighagen

**Affiliations:** 1 Ideaconsult Ltd., Sofia, Bulgaria; 10 in silico toxicology Gmbh (IST), Basel, Switzerland; 2 National Technical University of Athens, School of Chemical Engineering, Athens, Greece; 3 Karolinska Institutet, Stockholm, Sweden; 4 Douglas Connect GmbH, Zeiningen, Switzerland; 5 European Molecular Biology Laboratory – European Bioinformatics Institute (EMBL-EBI), Hinxton, United Kingdom; 6 Department of Analytical Chemistry and Computer Chemistry, University of Plovdiv, Plovdiv, Bulgaria; 7 Department of Bioinformatics, NUTRIM, Maastricht University, Maastricht, The Netherlands; 8 Computer Science Faculty, University of A Coruna, A Coruña, Spain; 9 IMT Institute for Advanced Studies Lucca, Lucca, Italy

**Keywords:** database, EU NanoSafety Cluster, nanoinformatics, nanomaterials, nanomaterials ontology, NanoQSAR, safety testing

## Abstract

**Background:** The NanoSafety Cluster, a cluster of projects funded by the European Commision, identified the need for a computational infrastructure for toxicological data management of engineered nanomaterials (ENMs). Ontologies, open standards, and interoperable designs were envisioned to empower a harmonized approach to European research in nanotechnology. This setting provides a number of opportunities and challenges in the representation of nanomaterials data and the integration of ENM information originating from diverse systems. Within this cluster, eNanoMapper works towards supporting the collaborative safety assessment for ENMs by creating a modular and extensible infrastructure for data sharing, data analysis, and building computational toxicology models for ENMs.

**Results:** The eNanoMapper database solution builds on the previous experience of the consortium partners in supporting diverse data through flexible data storage, open source components and web services. We have recently described the design of the eNanoMapper prototype database along with a summary of challenges in the representation of ENM data and an extensive review of existing nano-related data models, databases, and nanomaterials-related entries in chemical and toxicogenomic databases. This paper continues with a focus on the database functionality exposed through its application programming interface (API), and its use in visualisation and modelling. Considering the preferred community practice of using spreadsheet templates, we developed a configurable spreadsheet parser facilitating user friendly data preparation and data upload. We further present a web application able to retrieve the experimental data via the API and analyze it with multiple data preprocessing and machine learning algorithms.

**Conclusion:** We demonstrate how the eNanoMapper database is used to import and publish online ENM and assay data from several data sources, how the “representational state transfer” (REST) API enables building user friendly interfaces and graphical summaries of the data, and how these resources facilitate the modelling of reproducible quantitative structure–activity relationships for nanomaterials (NanoQSAR).

## Introduction

Nanotechnology is an increasingly dynamic area in materials science research and development, introducing novel materials with unique properties due to their size in the range of nanometres. A database and framework supporting nanomaterials safety has to comply with diverse requirements, set-up by the nanotechnology community. A number of challenges exist in the representation and integration of engineered nanomaterials (ENMs) data mainly due to the complexity of the data and the need to capture provenance.

### Physicochemical identity

The eNanoMapper framework must capture the physical and chemical identity of ENMs, including the notion of mixtures and their particle size distributions, differences in the amount of surface modification, manufacturing conditions and batch effects. It must also capture the biological identities (e.g., toxicity pathways, effects of ENM coronas, modes of action), interactions (cell lines, assays) and a wide variety of measurements. A number of analytic techniques have been proposed and developed to characterise the physicochemical properties of nanomaterials, including the commonly used dynamic light scattering to measure the particle size distribution and zeta potentiometry to estimate the pH-dependent surface charge.

### Biological identity

With the expanding insight into the factors determining toxicity, the list of measurable effects is growing increasingly long. The need for validated in vitro tests has been advocated since 2006 [1]. It is proposed to extend the list of endpoints for hazard identification to include cell uptake, cell viability, oxidative stress, inflammation, fibrosis, immunotoxicity, cardiovascular toxicity, ventilation rate, gill pathologies, mucus secretion and brain pathology. The EU guidance document lists the main known effects from experimental studies [2]. High-throughput omics data and kinetics [3] are becoming increasingly important in the assessment of nanomaterials, presenting challenges in both data management and analysis. A common requirement of all categories of users is to link the ENM entries with studies in which toxicology or biological interference of the nanomaterials have been studied, in addition to an accurate physicochemical characterisation.

### Data input, data formats, provenance, visualisation

The framework should allow for the representation of data and facts compatible with regulatory expectations and (inter)national standards. This usually translates into a set of available study summaries (rarely raw data) for a given ENM. The inclusion of links to product databases could also be considered (e.g., whether the nanomaterial occurs in nature, whether it is emitted by cars or is present in certain food sources, as well as known therapies in which the nanomaterial is used). However, supporting raw data files (including microscopy images) is an important requirement in contexts other than regulatory, enabling the reproducibility of the data preprocessing and analysis. Links to the corresponding protocols and data sources should be added, where available. Clear visualisation of nanomaterials that goes beyond just structural formulae should be available, in order to make the data less abstract biologists with less knowledge about nanomaterials.

### Support for data analysis

The modelling community presents a different requirement: the data analysis methods usually require a “spreadsheet” or matrix view of data for multiple ENMs. The experimental data in the public datasets are usually not in a form appropriate for modelling. Standardisation in these sources is specific to each database. Even in curated collections the preparation of data for modelling is not a straightforward exercise (e.g., the experimental values can be merged in many different ways into a matrix, depending on which experimental protocols and conditions are considered similar; also there could be multiple values due to replicates or similar experiments). The framework should allow for the addition of information based on the outcomes of the predictive toxicology models, including the biological role of the ENM, clearance, accumulation, and pathway information (e.g., WikiPathways entries [4]).

### Existing databases

Several databases exist that are relevant for ENM toxicity assessment. They list nanomaterials and a variety of their properties, or products containing nanomaterials: NanoMaterialRegistry (http://www.nanomaterialregistry.org/) [5], Nanoparticle Information Library NIL (http://nanoparticlelibrary.net/) [6], Nanomaterial-Biological Interactions Knowledgebase (http://nbi.oregonstate.edu/), caNanoLab (http://cananolab.nci.nih.gov/caNanoLab/) [7], InterNano (http://www.internano.org/), Nano-EHS Database Analysis Tool (http://icon.rice.edu/report.cfm), nanoHUB (nanohub.org/resources/databases/), NanoTechnology Characterisation Laboratory (http://ncl.cancer.gov/), EC JRC NanoHub (http://www.napira.eu/), the DaNa Knowledge Base (http://nanopartikel.info/) [8], and NanoWerks Nanomaterial Database (http://www.nanowerk.com/). The EU NanoSafety Cluster alone (http://www.nanosafetycluster.eu/) has many projects with database generating activities, such as NanoMiner [9]. An extensive review of existing nano-related data models, databases, and nanomaterials-related entries in chemical and toxicogenomic databases is presented in two recent publications [10–11]. Reviews of emerging databases and analysis tools in nanoinformatics have started to appear in the literature [12]. It becomes clear that nano-related data is relatively abundant, but also quite dispersed across many different sources. Combining data from various sources is hampered by the lack of programmatic access in most cases and the absence (or infrequent use) of suitable domain ontologies.

## Experimental

The eNanoMapper prototype database (http://data.enanomapper.net/) is part of the computational infrastructure for toxicological data management of ENM, developed within the EU FP7 eNanoMapper project [13]. It provides support for upload, search and retrieval of nanomaterials and experimental data through a REST web services API (http://enanomapper.github.io/API/) and a web browser interface. It is implemented by a customized version of AMBIT web services [14]. The database has been populated with content provided by project partners. We have recently described the design of the eNanoMapper prototype database [10] along with a summary of ENM data representation challenges and comparison to existing data models used to describe nanomaterials and assay data. The focus of this paper is the database functionality exposed through an application programming interface (API), and the use of the API for visualisation and modelling. While starting from the chemical compound-centric OpenTox API, the eNanoMapper prototype database implements a REST API, allowing for the representation of chemical substances with complex composition, and experimental data associated with those substances. The NMs are considered a special case of substances, which is consistent with the ontology representations, ECHA guidelines and peer-reviewed publications as elaborated in the next section.

### Chemical structures, substances, nanomaterials and measurements

The Nano Particle Ontology (NPO) defines a nanomaterial (NPO_199) as equivalent to a chemical substance (NPO_1973) that has as constituent a nano-object, nanoparticle, engineered nanomaterial, nanostructured material, or nanoparticle formulation. Chemical substances are classified as types of chemical entity (NPO_1972). The default approach for representation of chemical compounds in ISA-Tab [15] is an ontology entry, which typically points to a single chemical structure. This is insufficient for describing substances of complex composition such as nanomaterials, hence a material file was introduced to address this need in ISA-Tab-Nano [16]. The latest ISA-Tab-Nano 1.2 specification recommends using the material file only for material composition and nominal characteristics, and to describe the experimentally determined characteristics in regular ISA-Tab assay files. The definitions of the terms “substance” and “material” are discussed in [17], comparing ISO, REACH and general scientific definitions of the terms. The REACH definition of a substance encompasses all forms of substances and materials on the market, including nanomaterials; a substance may have complex composition. The paper [17] notes that the OECD Harmonized Templates (OHT) definition of “reference substances” is very similar to the definition of the term “reference material”. The same publication refers to the “test” and “measurement” terms as the fundamental concepts [17]. The OECD guideline defines the “test” or “test method” as the experimental system used to obtain the information about a substance. The term “assay” is considered a synonym. The term “testing” is defined as applying the test method. The endpoints recommended for testing of nanomaterials [18] by the OECD Working Party on Manufactured Nanomaterials (OECD WPMN) use the terms and categories from the OECD Harmonized Templates. The NPO distinguishes between the endpoint of measurement (e.g., particle size, NPO_1694) and the assay used to measure the endpoint (e.g., size assay, NPO_1912), where the details of the assay can be further specified (e.g., uses technique electron microscopy, NPO_1428). This structure is generally the same as the one supported by the OHT (e.g., in the OHT granulometry type of experiment several size-related endpoints can be defined, as well as the equipment used, the protocol and specific conditions). The CODATA UDS [19–20] requires specification of how each particular property is measured. ISA-Tab-Nano also allows for defining the qualities measured and detailed protocol conditions and instruments. The level of detail in the OHT, CODATA UDS, ISA-Tab-Nano and available ontologies differ, which is due to their different focus. Mapping between terms defined in the different sources is an ongoing effort supported by the eNanoMapper ontology team and the EU NanoSafety Cluster database working group. In Supporting Information File 1, we provide a table of OECD WPMN recommended endpoints and their potential correspondence to UDS and ISA-Tab-Nano concepts.

To summarise, the most important data objects necessary to represent nanomaterials and NM characterisation are the substance with its composition, and a data object, able to represent a test method, its application to the substance under specific conditions and the measurements obtained as a result of this process. Therefore, the objects supported by the API are “substances” (as a superclass of nanomaterials), “protocols”, “endpoints”, “conditions”, “protocol applications” and “measurements”. A “protocol application” (a term borrowed from ISA-Tab) explicitly describes a single step of the experimental graph, namely the application of a particular protocol with its specific parameters to the source material and includes the corresponding results (be it a sample or data readouts). For the purposes of ENM database integration, the source material is always a chemical substance (ENM) with its composition and linkage, while the result is a set of measurements, each annotated with the relevant endpoints and experimental conditions. While we support importing files generated from IUCLID5 database and thus all OECD WPMN recommended endpoints, the list of endpoints in the database is not fixed, and arbitrary endpoints can be imported through spreadsheets and further annotated with ontology entries. The measurement can be specified by a value, range of values, error measure and units, or by a link to a raw data file (e.g., an image). This representation directly supports the OHT data model, and the notion of a set of measurements is very similar to the measurement group concept in the Bio Assay Ontology (BAO) [21], as well as encompassing the measurement value concept in the CODATA UDS. In order to support raw data, we decided to extend the measurement value beyond scalar values and include links to measurement artifacts, such as image and raw data files, similarly to the ISA-Tab approach. The ability to describe derived measurements, by linking measurement groups, as supported by BAO and implied in UDS, is currently being considered, especially in order to support the modelling activities in eNanoMapper. The data model is sufficiently flexible to represent scenarios like multiple endpoints readouts within a single experiment, dose response data as well as replicated measurements. Examples are shown in the visualisation section.

### Ontology

The eNanoMapper strategy to adopt and extend ontologies in support of data integration has recently been described [22]. eNanoMapper supports ontology re-use, for example it re-uses the content of the NPO and BAO, through automated modular import of content subsets into an integrated whole. However, the scope of the ontology goes beyond any of the individually imported ontologies, encompassing the whole of the domain of nanomaterial safety assessment. The strategy of re-use of existing ontology content enables downstream annotated data in different repositories to be integrated wherever the same identifiers are used in annotation. The ontology is available at http://purl.enanomapper.net/onto/enanomapper.owl, from BioPortal at http://bioportal.bioontology.org/ontologies/ENM, and for download in full from the development repository on GitHub (https://github.com/enanomapper/ontologies). This section describes the strategy for application of the ontology to the annotation of the prototype eNanoMapper database content.

All data in the database is targeted for annotation with relevant ontology entries from the composite eNanoMapper ontology. Each entry in the ontology has a unique IRI (International Resource Identifier), for example “nanomaterial” (a class imported from the NPO) has the IRI http://purl.bioontology.org/ontology/npo#NPO_199. The IRI consists of an ontology namespace as prefix, followed by a unique identifier for the particular term. For brevity, throughout this manuscript we have referred simply to ontology identifiers (IDs) without the full IRI including the prefix. However, expansion from the short ID to the full IRI is a deterministic transformation. Classes are also associated with a unique label and a descriptive textual definition. The IRI, based on the same underlying Semantic Web technology as the eNanoMapper database prototype, offers a semantics-free stable identifier that is suitable for use in data annotation, as it is resistant to minor changes in the label and improvements in the definition of the class.

**Figure 1:**
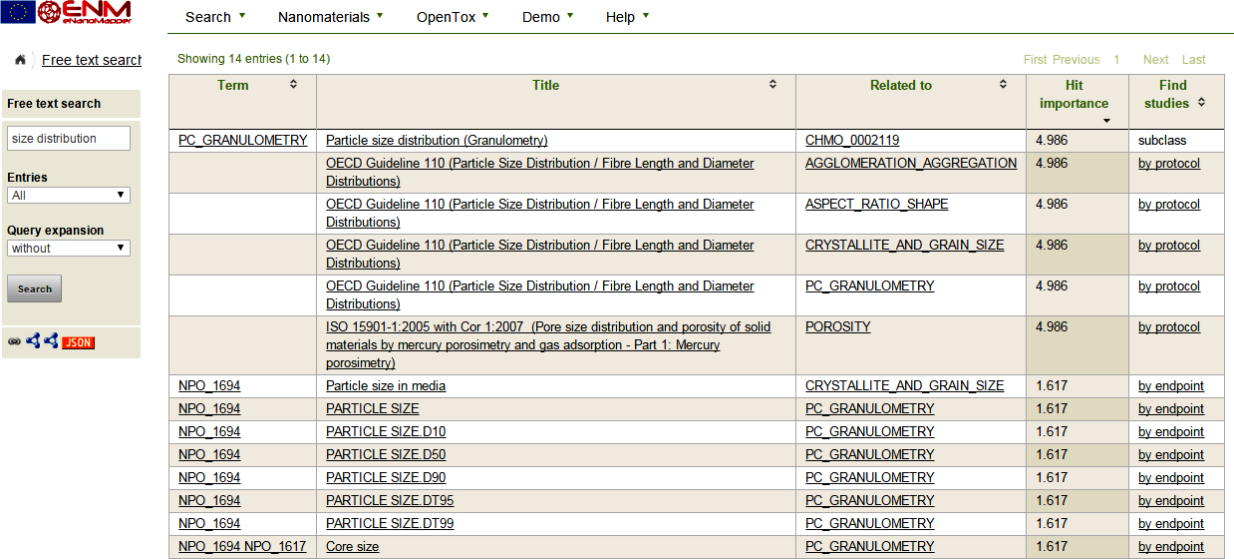
Screenshot illustrating free text search finding ontology annotated database entries (e.g. protocols and endpoints in the second column). The last column is a link leading to a list of studies.

Examples of annotations that have already been included in the database are: “particle size distribution (granulometry)” annotated to the ID CHMO_0002119 in the Chemical Methods Ontology namespace, “aspect ratio” annotated to the ID NPO_1365 and “shape” to ID NPO_274 in the NPO namespace (Figure 1).

Annotations are selected from the available classes in the eNanoMapper ontology; a best match approach is used which aims to select the most specific class available for annotation. When no suitable class is present, a suitable class may be found in the broader BioPortal collection which is then targeted for inclusion in the eNanoMapper ontology. If no suitable class exists even within the full collection of ontologies in BioPortal, a request is issued for the class to be added in the eNanoMapper ontology manually. We formally document all such requests via our public GitHub issue tracker (https://github.com/enanomapper/ontologies/issues). Once the term has been included in the ontology it is released to the wider community and becomes available in tools such as BioPortal automatically.

The hierarchical classification structure of the ontology, together with the use of domain-specific relationships, is envisioned to enable intelligent searching, browsing and clustering tools to be developed in the future, as well as to enable templates to be implemented for database content entry compliant with Minimum Information guidelines.

### Application programming interface (API)

The eNanoMapper architecture has been informed by the prior experience of several of the authors in designing and building the OpenTox predictive toxicology framework for chemicals [23] and their involvement in developing and supporting the ToxBank [24] data warehouse for the SEURAT-1 research cluster [25]. The framework design adopts the REpresentational State Transfer (REST) software architecture style, a common information model that supports ontology annotation, and an identity service and an access control based on OpenAM [26]. The REST architecture can be briefly summarized as being composed of a collection of information entities (resources), in which each entity can be retrieved by its address and supports a limited number of operations (e.g., read and write). The overall system architecture of eNanoMapper extends the OpenTox [23] and ToxBank [24] designs. Both consist of a set of web services that provide access to experimental protocols, raw and processed data, and data analysis tools. The web services do not need to be deployed on the same machine, but can also be distributed on independent servers. Communication through well-defined interfaces facilitates adding new services, such as services that support new data types or search functionality. The eNanoMapper API is documented online using the Swagger (http://swagger.io/) specification, accessible as interactive documentation at http://enanomapper.github.io/API/.

#### Substance resource

**Figure 2:**
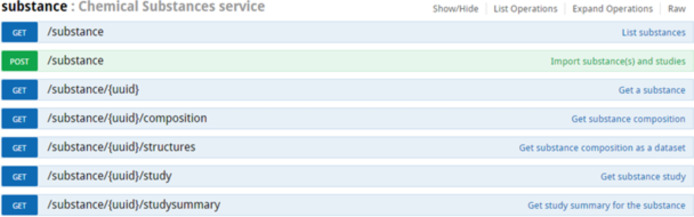
Top level substance API documentation. The “GET /substance” call is used to retrieve or search a list of NM, subject to multiple query parameters defining the NM search. The “POST /substance” call is used to upload NM and study data in supported formats. The “/substance/{uuid}” call is used to retrieve the substance specified by its unique identifier. Each substance is identified with an unique identifier, generated or specified on import in the form of UUID. The rest of the calls allow to retrieve the component of the NM, the study data and a summary of the available data for the NM, grouped by endpoints.

While the OpenTox framework is intentionally centred on chemical compounds, eNanoMapper uses an extension, allowing representation of chemical substances with a defined composition (Figure 2) and experimental data, associated with substances, rather than associated with chemical structures.

**Figure 3:**
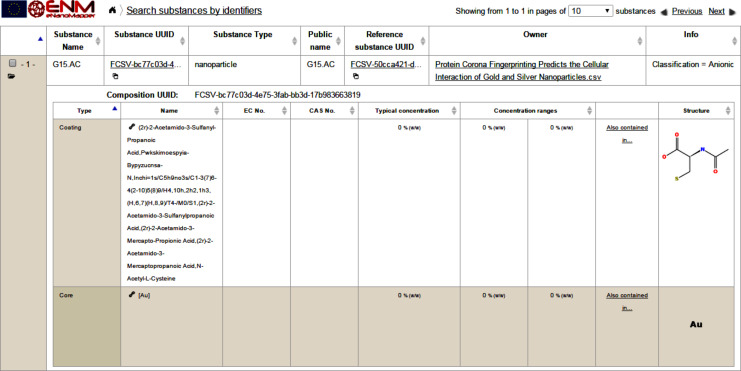
Screenshot showing a nanomaterial entry (a gold nanoparticle with the name G15.AC) and its components (a gold core and organic coating). The components can be retrieved through the “/substance/{uuid}/composition” API call and are linked to the OpenTox API compound resources, which allows for the execution of chemical structure based calculations and predictions. This NM entry is part of the the Protein Corona dataset described below and was imported via a spreadsheet (.csv) file. The “reference substance UUID” refers to the chemical structure, which is considered the main component (Au in this case). The “Owner” column typically refers to the NM manufacturer, or if such information is missing it refers to the data file used for import. The “Info” column may contain an arbitrary key-value data, typically referring to the NM identifiers in other systems.

The substance resource supports assigning a nanomaterial type, a chemical composition with relevant concentration and constituents roles, and links to the OpenTox compound resources for specifying the chemical structure, where relevant. NMs are considered a special case of substances. Figure 3 shows the eNanoMapper prototype database user interface displaying the components of a gold nanoparticle with an organic coating. The visualisation is implemented as a JavaScript widget, which consumes the substance API.

**Figure 4:**
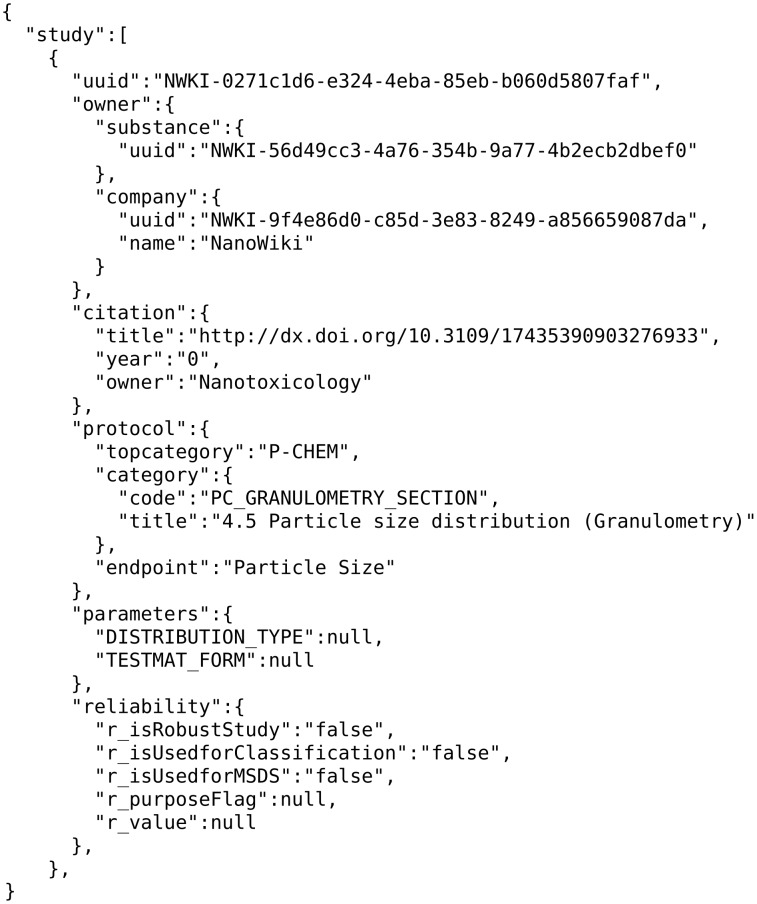
Experimental data JSON example.

The experimental data are assigned to a substance (e.g., nanoparticle) and a JSON (JavaScript Object Notation) representation of the data can be retrieved through a “/substance/{uuid}/study” API call. As an example, in Figure 4, we present an excerpt from the JSON serialisation of a cell viability assay for the NanoWiki [27] entry with identifier *NWKI-56d49cc3-4a76-354b-9a77-4b2ecb2dbef0*, retrieved from https://apps.ideaconsult.net/enanomapper/substance/NWKI-56d49cc3-4a76-354b-9a77-4b2ecb2dbef0/study.

**Figure 5:**
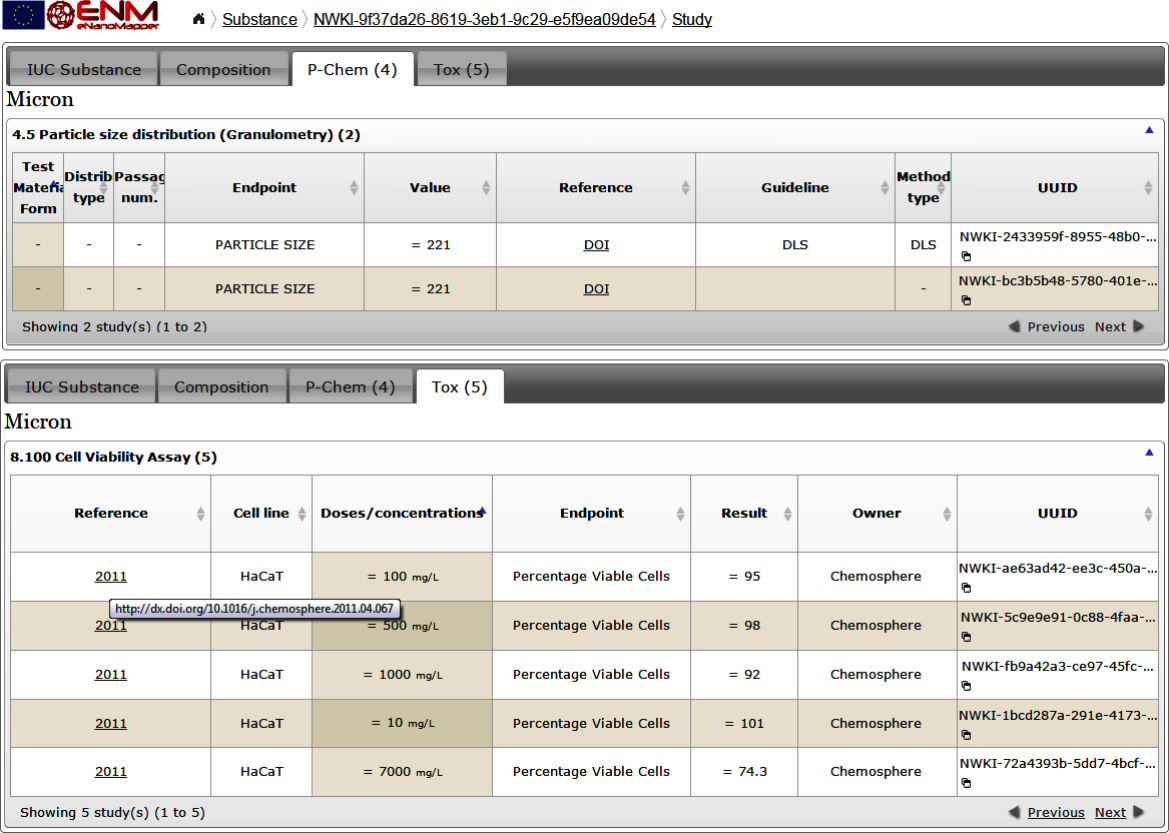
Physico-chemical and toxicity data from the NanoWiki data set.

Similarly to the nanoparticle composition shown in Figure 3, the visualisation of physico-chemical and biological data (Figure 5) is implemented as a JavaScript widget, consuming the substance API.

#### Search

**Figure 6:**
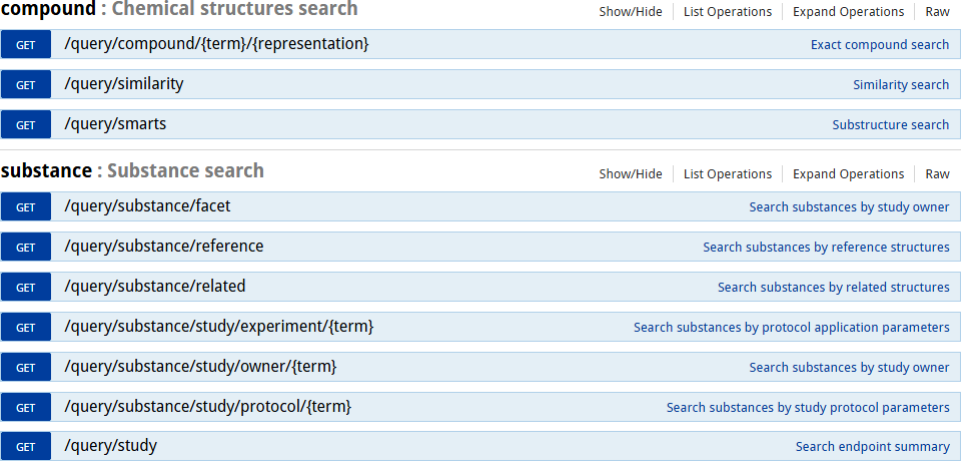
Compound, substance and study search API documentation.

The API offers access to a variety of searches by substance identifier, any combination of measurement endpoints, and/or chemical structure (Figure 6). The JSON serialisation is the same as above, screenshots of the currently implemented user interface are shown in the Results section.

#### Data import

**Figure 7:**
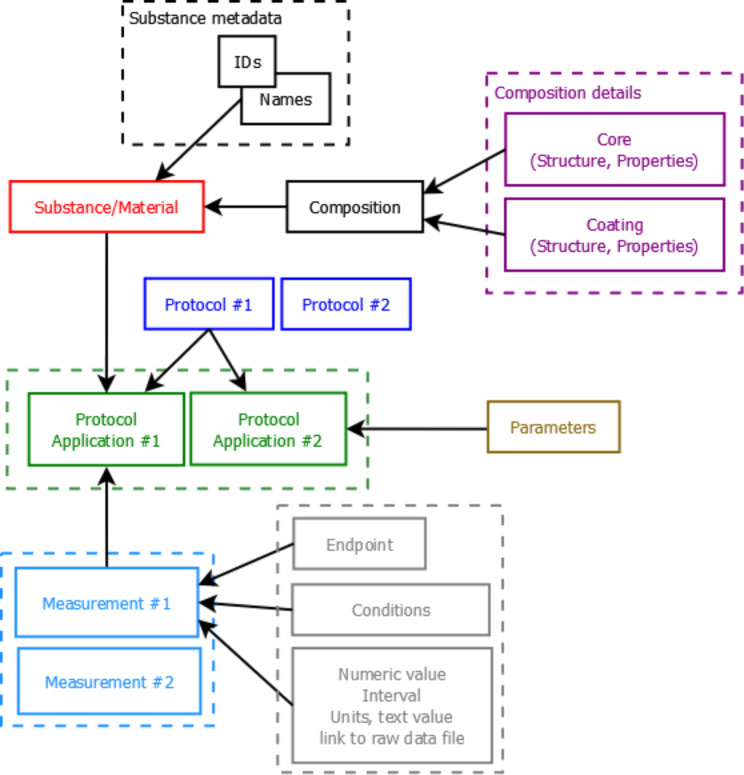
Outline of the data model: Substances are characterised by their “composition” and are identified by their names and IDs. The event of applying a test protocol to a substance/material is described by a “protocol application” entity. Each protocol application consists of a set of “measurements” for a defined “endpoint” under given “condition”. The measurement result can be a numeric value with or without uncertainty specified, an interval, a string value, or a link to a raw data file (e.g., a microscopy image).

The data model (Figure 7) allows for integration of content from a variety of sources, namely OHTs (IUCLID5 .i5z files or direct retrieval of information from IUCLID5 servers, http://iuclid.eu/); custom spreadsheet templates (e.g., Protein Corona CSV files or ModNanoTox Excel files), and custom formats, provided by partners (e.g., the NanoWiki RDF dump [27]). ISA-Tab [15] files are converted by compressing the chain of protocols into a single entry, yet retaining all the protocol parameters and recording the material as a substance and the rest of the factors as experimental conditions. The NanoWiki RDF dump is converted with a custom parser. The supported import formats are currently being extended to include ISA-Tab-Nano [16] and a large set of custom spreadsheet templates.

Taking into account the observation that the use of spreadsheet templates is the preferred approach for data entry by the majority of the EU NanoSafety Cluster projects, we developed a configurable spreadsheet parser facilitating user friendly data preparation and upload. The parser enables import of the data, stored in the supported set of spreadsheet templates, and accommodates different row-based, column-based or mixed organizations of the data. The parser configuration is defined in a separate JSON file, mapping the custom spreadsheet structure into the internal eNanoMapper storage components: “Substance”, “Protocol”, “Measurement”, “Parameters” and “Conditions”. The JSON configuration syntax includes a set of keywords, specifying different strategies for reading the data from one or several sheets, as well as allowing combination of the excel structures (sheets, rows, columns, blocks of cells and cells) into the eNanoMapper data model. The parser code, the JSON syntax, documentation and example files are available at https://github.com/enanomapper/nmdataparser/. The mapping enables a uniform approach towards import, storage and searching of the ENM physicochemical measurements and biological assay results. While the parser itself is open source, the configuration files may not be, thus not revealing the organisation of confidential data templates. The parser is currently being used to parse ModNanoTox templates and confidential templates from EU NanoSafety Cluster projects. Maps of the confidential spreadsheet templates are available on request, in compliance with the agreements between the corresponding projects. More formats will be supported as needed for indexing data from different sources. The development of ISA-Tab-Nano and RDF import and export tools is ongoing.

**Figure 8:**
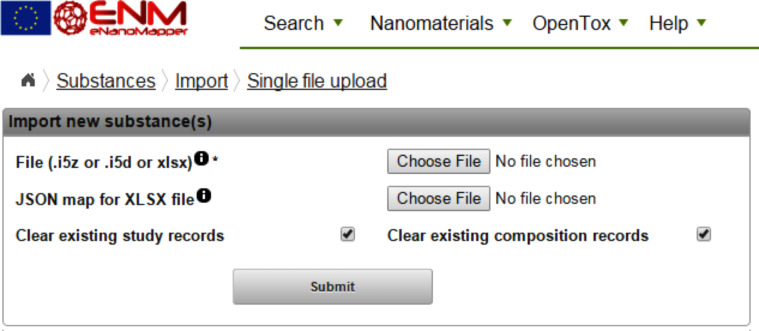
Data upload web page of the database system showing support for two file formats.

The data import is performed by HTTP POST to the substance resource (Figure 2), which translates to a regular web form for file upload (Figure 8). The two checkboxes control whether the composition records and study records for the materials being imported will be cleared, if already in the database. Each material entry in the database is assigned a unique identifier in the form of a UUID. If the input file is *.i5z or *.i5d, the identifiers are the IUCLID5 generated UUIDs already present in these files (e.g., IUC5-5f313d1f-4129-499c-abbe-ac18642e2471). If the input file is a spreadsheet, the JSON configuration defines which field to be used as an identifier and uses the field itself or generates UUID from the specified field (e.g., FCSV-bc77c03d-4e75-3fab-bb3d-17b983663819 indicates the entry imported from CSV file). The parser may be configured to use a custom prefix on import, e.g., ”NWKI-” for NanoWiki entries, generating UUID like ”NWKI-71060af4-1613-35cf-95ee-2a039be0388a”.

#### Datasets of substances (bundles)

**Figure 9:**
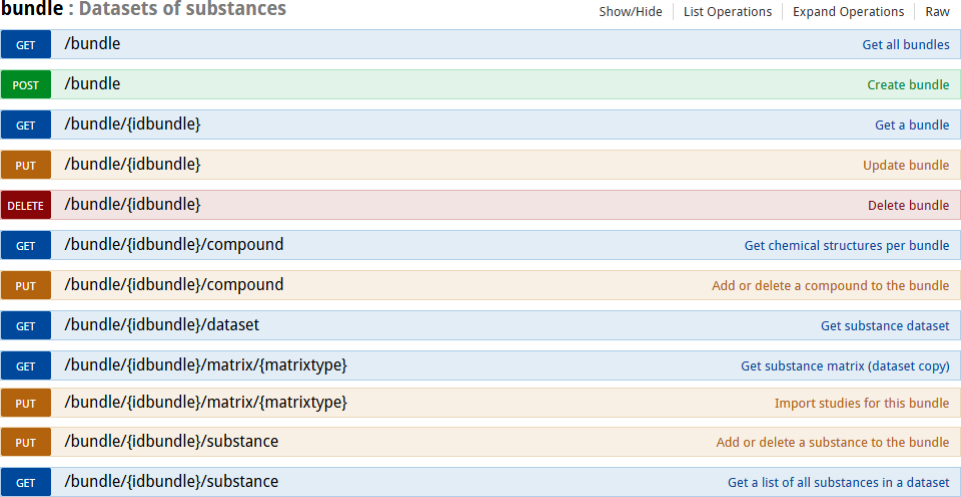
Bundle API documentation at http://enanomapper.github.io/API. A bundle is a REST resource, allowing one to retrieve all information about a selected set of NMs and endpoints by a singe REST call. The PUT calls allow one to select or deselect the NMs and the endpoints.

**Figure 10:**
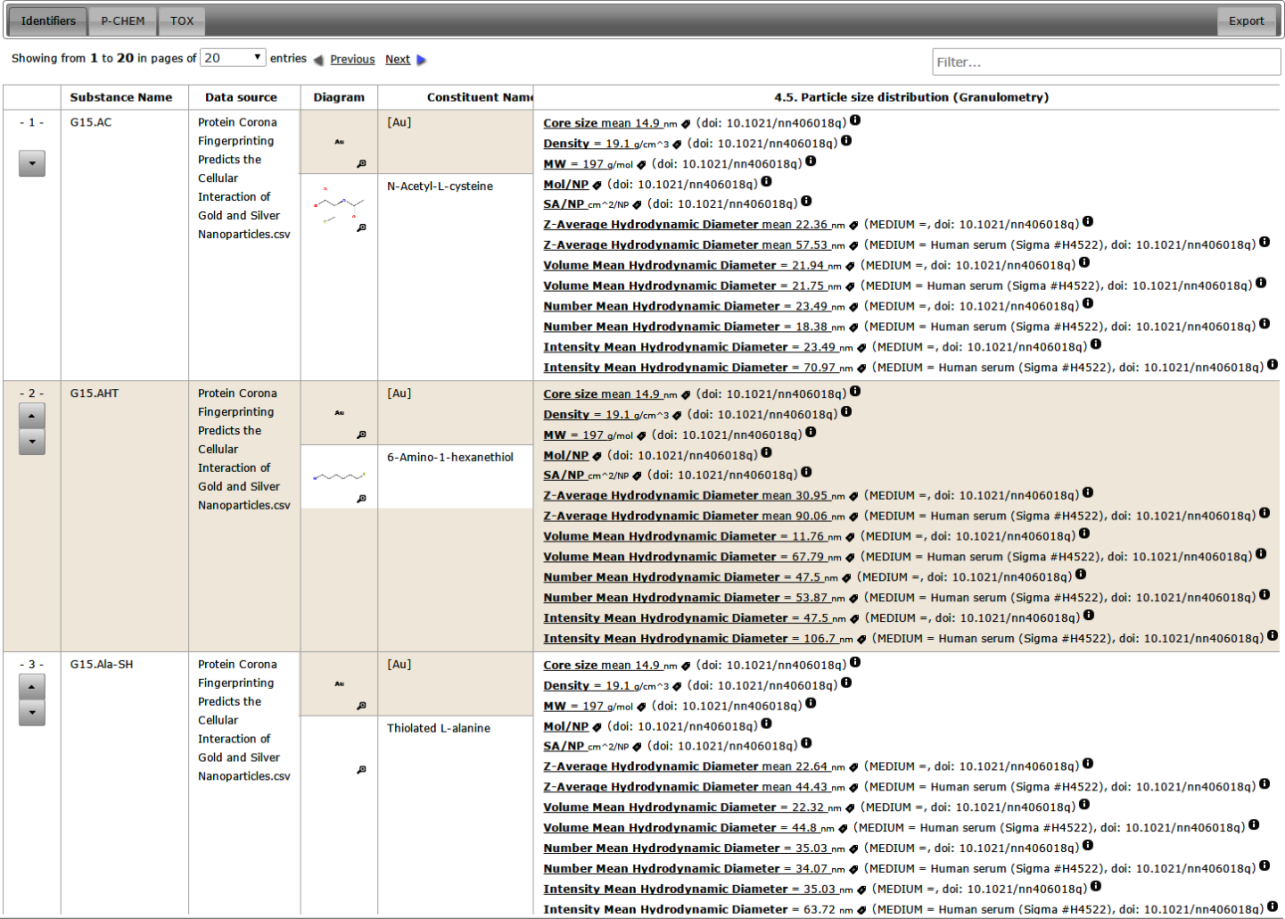
Screenshot of the bundle view with the Protein Corona data set. In addition to the Substance API, which allows one to retrieve study data for a single NM as in Figure 5, the bundle API provides efficient means to retrieve information about a set of NMs.

A “bundle” (Figure 9) is a REST resource that groups a selected set of substances and a selected set of endpoints. This functionality was introduced to enable creating groups of diverse nanomaterials, to specify the endpoints of interest, which can vary from physicochemical to proteomics assays, and to enable retrieving all this data with a single REST call. A bundle may include the nanomaterials and assay data from a single investigation as well as serve as a container for a set of NMs and for data (typically representing different experiments) retrieved from the literature. The latter is currently difficult to achieve in ISA-Tab, as its purpose is to capture the experimental graph of a single investigation. The bundle API can be considered an extension of the original OpenTox compound-centric dataset concept to allow for datasets of nanomaterials. The experimental values may include replicates and range values and can be merged in many different ways into a matrix (Figure 10), depending on which experimental protocols and conditions are considered similar. The API in Figure 9 provides one of many possible ways of conversion into a matrix form through the “/bundle/{id}/matrix” call. The users can build external applications, retrieving the experimental data and applying custom conversion procedures, as does the Jaqpot Quattro application described in the “Modelling” section.

## Results

The results include using the eNanoMapper database described above to import and publish online ENM and assay data from several sources; as well as the demonstration of how the REST API enables building a user friendly interface and graphical summaries of the data, and last but not least, facilitates reproducible Quantitative Structure Activity Relationship for nanomaterials (NanoQSAR) modelling.

The demonstration data provided by eNanoMapper partners – (i) NanoWiki, (ii) a literature dataset on protein coronas and (iii) the ModNanoTox project dataset – illustrates the capability of the associated REST API to support a variety of tests and endpoints, as recommended by the OECD WPMN.

### NanoWiki

NanoWiki was originally developed as an internal knowledge base of the toxicity of, primarily, metal oxides at the Karolinska Institutet and Maastricht University. The database is developed as a wiki using the Semantic MediaWiki platform, running on a virtual machine using the VirtualBox software. The wiki contains physicochemical properties and toxicological data for more than three hundred nanomaterials: more than two hundred metal oxides, 80 carbon nanotubes, and a few metal and alloy particles. All nanomaterials originate from data in 34 papers, identified by Digital Object Identifier (DOI), from twenty scientific journals. Because the amount of physicochemical detail differs from one paper to another, each material is characterized with different measured characteristics. Each measurement may have a single value (median or average, though this is not always specified), a minimum and maximum value, or a single value and a standard deviation. Biological measurements are linked to assays (such as cytotoxicity, cell growth, cell viability, genotoxicity, and oxidative stress), endpoints measured on that assay (e.g., ROS concentration, GI50, percentage viable cells), and cell line information, though not consistently.

Importing the data into eNanoMapper takes advantage of NanoWiki using Semantic MediaWiki and its template framework: all data relevant to NanoQSAR can be retrieved from the wiki as RDF, in the form of a RDF/XML data dump [27] (in addition to the common MediaWiki XML and SQL dumps of the wiki content).

### ModNanoTox

The ModNanoTox EU FP7 project (http://www.birmingham.ac.uk/generic/modnanotox/index.aspx) has produced a survey and selection of relevant physicochemical properties to use towards building a range of descriptors of engineered nanoparticles (mainly metal-based) and their potential toxicity. This dataset nicely demonstrates the complexity of the nanosafety domain. The ModNanoTox database provides physicochemical descriptors and toxic activities of nanoparticles from several studies. The database version from August 2013 includes 86 assays with more than 100 different endpoints affecting 45 species.

Unfortunately, only a few nanoparticles (usually fewer than three) have been tested for each endpoint. Physicochemical descriptors for the characterisation of nanoparticles are incomplete as well (about 75% missing values). The two most comprehensive species in the dataset are *Daphnia magna* (water flea) and *Danio rerio* (zebrafish), with 34 and 14 assays each. The best represented endpoint for Daphnia is “Mortality”, and we were able to extract about forty “LC50” and sixty “% survival” data entries. In both cases the number of measured nanoparticle properties was very low. Most studies report only two to four different nanoparticle properties (descriptors) and the descriptor types are very inconsistent (overall 36 different descriptors, which results in very sparse matrices with a high number of missing values).

The ModNanoTox data import is currently being tested and is not yet available online. The ModNanoTox data set was provided as a MSExcel spreadsheet file. It consists of four sheets describing, respectively, (i) investigation study details, (ii) particle details and physicochemical properties, (iii) assay protocol description and (iv) assay measurement outcomes. The information in all sheets is organized as a sequence of dynamic blocks of data, each one containing a variable number of rows. The configurable spreadsheet parser described in the “Data Import” section supports the recognition of blocks and the synchronization between blocks within the four sheets. The next step is to divide the data in each block into groups and sub-groups and match them across the sheets. This last operation is implemented by a dedicated command line application, built on top of the configurable data parser and allowing parsing of the entire ModNanoTox complex organisation into the internal eNanoMapper data model.

### Protein Corona

The demonstration data set, extracted from [28], focuses on the biological identity of ENMs. The authors used the composition of the protein corona “fingerprint” to predict the cell association of a 105-member library of surface-modified gold nanoparticles (see Figure 3). 785 distinct serum proteins were identified by LC-MS/MS, from which 129 were suitable for relative quantification. The fingerprint of serum proteins was defined by the relative abundance of each protein on a nanoparticle formulation. The value of individual proteins within the serum protein fingerprint for predicting cell association was explored by the authors by developing a series of log-linear models that model the influence of the relative abundance of each adsorbed serum protein on net cell association. Among the factors in play in protein corona, biological interaction was chosen to be represented by cell association because of its relevance to biodistribution, inflammatory response potential, and in vivo toxicity. The eNanoMapper prototype described in this paper is able to capture this protein corona, and modelling approaches were extracted from these data for statistical analysis.

### Data quality considerations

While there is a common agreement on the importance of data curation, there is no well established common understanding of how it should be performed. Approaches range from simple data cleaning to the entire spectrum of data-related activities including evaluation, on-going data management, and added value provisioning through analytic tools. The focus of this publication is on the data management system, allowing for a unified approach to storage and querying of NM related data. Using the data for modelling and being able to write the prediction results back is only one of the possible ways to add value. Future developments may include providing support for emerging paradigms such as Adverse Outcome Pathways [29], categorization strategies via decision trees [30] and principal components [31]. We intentionally do not discuss data evaluation and clean-up for the following reasons. Firstly, at present we are not aware of universally adopted criteria for evaluation of NM data, although there are a number of related activities in the EU NanoSafety Cluster projects and worldwide, as well as specific sets of rules implemented in existing databases such as the NanoMaterial Registry (https://www.nanomaterialregistry.org/about/WhatIsCuratedData.aspx). In regulatory toxicology the Klimisch codes [32] are the accepted approach, enforced in Europe by the relevant guidance [33] and the IUCLID database. They provide definitions and support for annotating the data records by relevance, reliability and adequacy. Some of the criteria necessarily overlap with rules defined elsewhere (availability of the raw data, adequate description of the study, protocols, parameters, purities/impurities and the origin of the test substances; proof of ability of the lab to do the study). Klimisch codes (or scores) define four reliability categories (1 = reliable without restrictions, 2 = reliable with restrictions, 3 = not reliable, 4 = not assignable), where score 1 or 2 can only be assigned if the data are generated through accepted standard methods (e.g., OECD guidelines or equivalent national or international standards) and according to Good Laboratory Practice (GLP). In practice, very few of the publicly available NM datasets can be assigned reliability code 1 or 2, due to the lack of standard or validated protocols, deviations, or just an absence of details. The criteria for experimental protocol validation are out of scope for this paper as well as for the eNanoMapper project. However, the database and import templates are designed to require that the test protocol be specified for every data entry. Secondly, as the goal is to support data originating from different sources and typically already having undergone some kind of evaluation and assigned relevant labels, the most appropriate way is to import the data as it is and keep the original quality labels. For example the OECD HT templates do include fields for Klimisch scores and the eNanoMapper database does store these scores, as is shown in the JSON serialization. The data generated or gathered from the literature by EU NanoSafety Cluster projects have already been evaluated as part of these project activities, and we intend to keep this information, where it is available. Once the data are converted into the common data model, rules checking the presence or absence of raw data, protocols, deviations, and parameters can be applied automatically, which is a more efficient approach than checking these rules manually before import. The ontology annotation might help to overcome some of the challenges, such as different evaluation criteria and different terminology for the quality labels. In cases where automatic tools fail, working closely with data providers to improve the quality and gain common understanding of the data is necessary. This approach is also in line with the intention “not to exclude automatically the unreliable data from further considerations” [32] and that “there is unlikely to be a single out-of-the-box solution that can be applied to the problem of data curation. Instead, an approach that emphasizes engagement with researchers and dialogue around identifying or building the appropriate tools for a particular project is likely to be the most productive” [34].

### Visualisation

#### User interface

**Figure 11:**
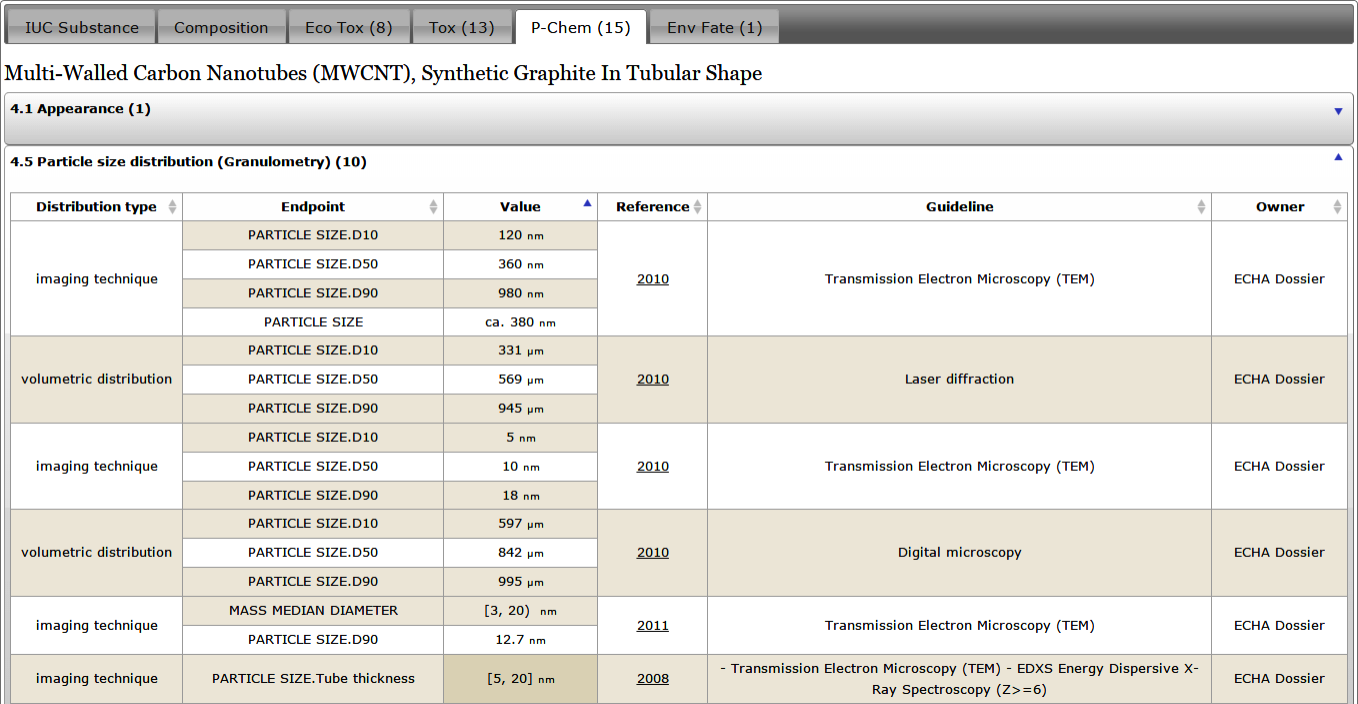
Physicochemical data for multi-walled carbon nanotubes. The screenshot illustrates the data model and UI support for size distribution (through percentiles D10, D50, D90), multiple endpoints per measurement (Mass median diameter and particle size), and multiple experiments using different protocols.

**Figure 12:**
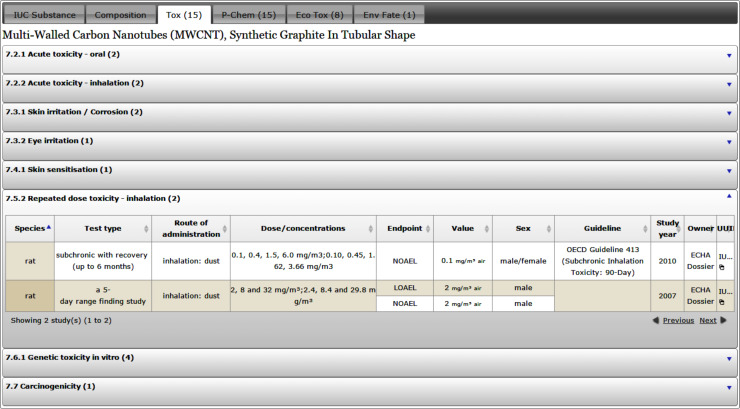
Toxicity data for multi-walled carbon nanotubes. The repeated dose toxicity (inhalation) is shown in the expanded row, illustrating support for multiple endpoints (LOAEL, NOAEL) and test types.

The following screenshots illustrate the eNanoMapper prototype database user interface, as implemented by AMBIT web services [14], with the help of JavaScript widgets consuming the REST API. The screenshots in Figure 11 and Figure 12 illustrate the data model support and the visualisation of experimental data, consisting of a variety of endpoints, experimental conditions and multiple endpoints values. The origin of the data is the ECHA dissemination site [35], and the data were manually entered into a local IUCLID5 instance, exported into IUCLID5 .i5z file and imported into the database.

The API is tightly integrated with a chemical structure and chemical similarity search (implementation details previously published in [14,36–37]). Chemical similarity is a pivotal concept in cheminformatics, encompassing a variety of computational methods quantifying the extent to which two chemical structures resemble each other. Apart from the “intuitive notion” of chemical similarity typically acquired during chemistry education, the computational methods vary from structure-based (2D, 3D), descriptor- and field-based approaches [38]. Chemical similarity evaluation requires two components, namely a numerical representation of the chemical structure and a measure allowing for comparing two such representations. The representations derived from the molecular graph are by far the most common (e.g., hashed fingerprints and various flavours of substructure keys) and the Tanimoto coefficient is the most popular similarity measure. The chemical similarity values usually range from zero (no similarity) to one (identical structures). Similarity searching (along with chemical substructure searching) in chemical databases is considered standard functionality and is nowadays offered by all state-of-the-art chemical databases and cheminformatics tools [39].

**Figure 13:**
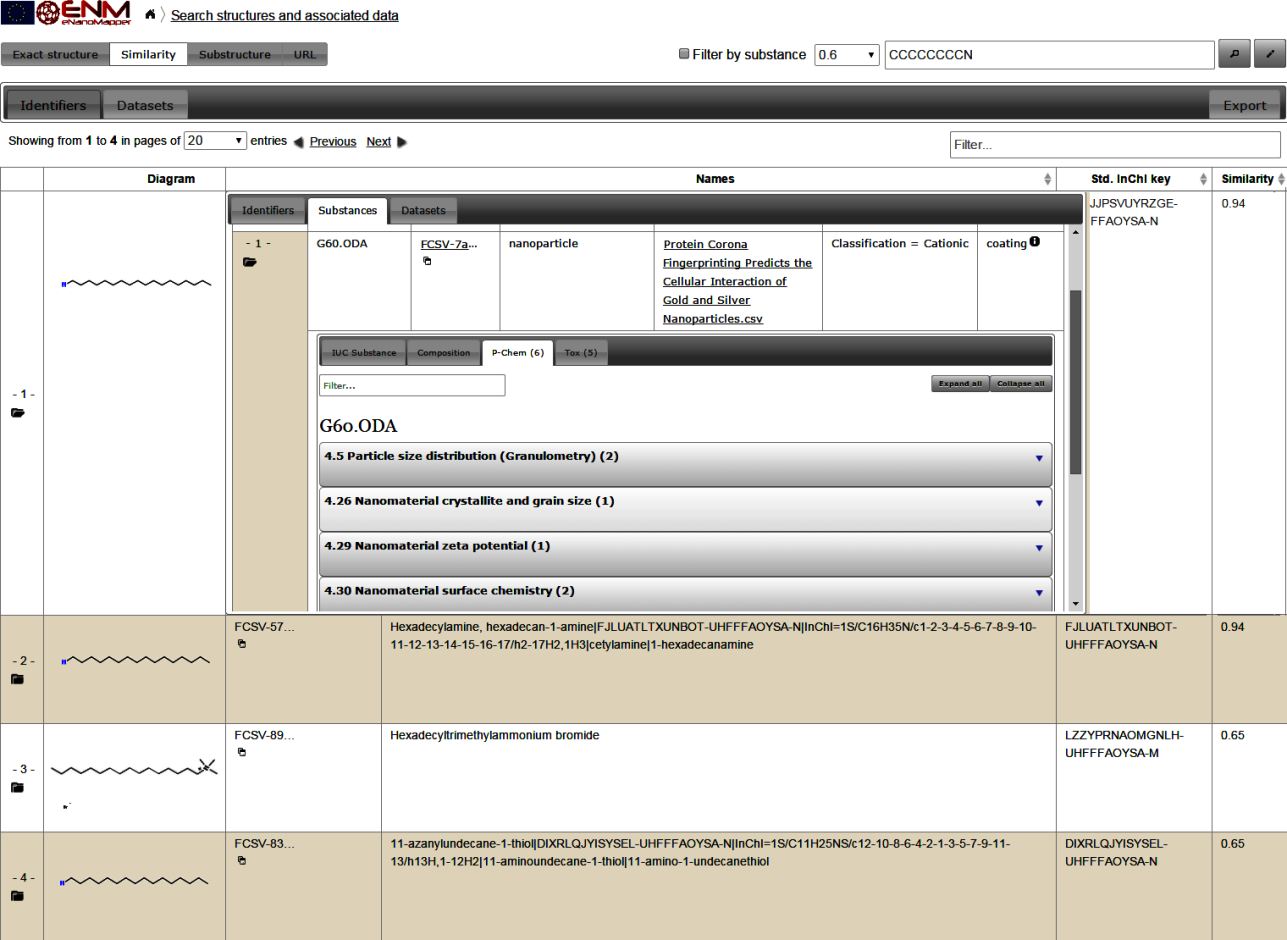
Screenshot showing the results of a chemical similarity query (octyl amine, SMILES CCCCCCCCN) with a similarity threshold Tanimoto coefficient = 0.6. The results include octadecylamine (similarity 0.94), hexadecylamine (similarity 0.94), hexadecyltrimethylammonium bromide (similarity 0.65), 11-amino-1-undecanethiol (similarity 0.65), all used as coating of silver and gold nanoparticles in the protein corona dataset. The first row shows expanded view with details of the NM.

The chemical similarity search in the eNanoMapper prototype database enables querying by a chemical structure of a NM component and highlighting the results as a core, coating or functionalisation component (Figure 13). The reason for the wide adoption of the similarity approach is the assumption of the “similarity property principle” or “neighbourhood behaviour”, namely that “similar compounds should have similar properties”. This principle puts the chemical similarity at the core of methods and tools supporting property prediction, structure–activity relationship, chemical database screening, virtual screening in drug design, and diversity selection. The similarity assessment based on structure analogy is the basis of read across and chemical grouping. However, there is a common understanding that the most difficult part in read across is “rationalising the similarity”. Violations of the “similarity property principle” exist due to a variety of reasons [38], and nowadays the existence of “activity cliffs” (small changes in the chemical structure leading to a drastic change in the biochemical activity) is well known. A recent review by Maggiora [40] outlines the methods used as well as the pros and cons of using the molecular similarity framework in medicinal chemistry. In the context of nanosafety assessment there is not yet a standardized approach for NM similarity, however a number of attempts for NM grouping and read across have been published recently [41–42].

**Figure 14:**
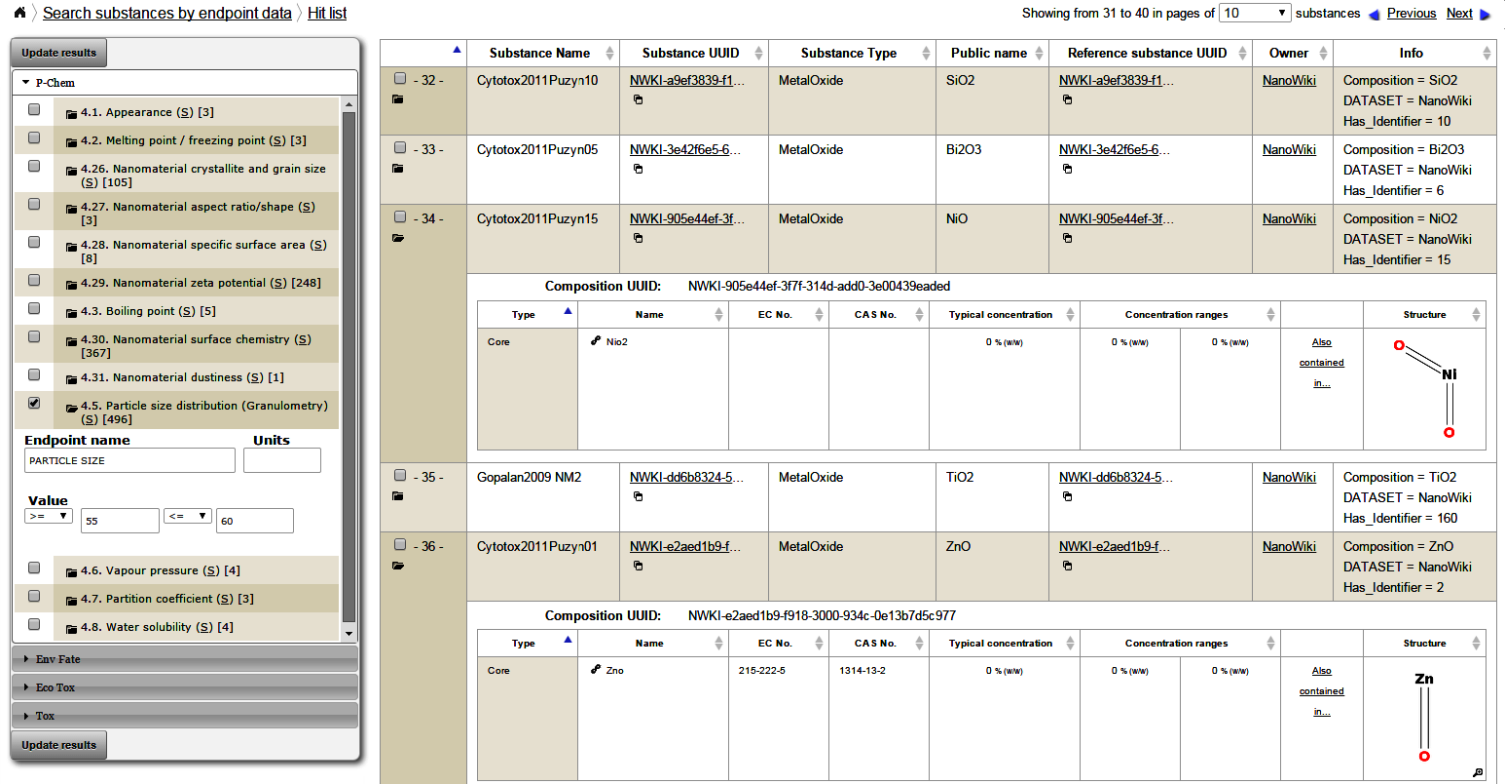
Screenshot showing query results in the NanoWiki data set for particle sizes between 50 and 60 nm. The widget at the left side represents an overview of all experimental data in the system, organized in four groups of physicochemical, environmental, ecotoxicological and toxicity sections. Each section lists available endpoints and the number of available data entries. The text boxes support auto-completion, i.e., the available values will be displayed and can be selected by either pressing an arrow-down button (to list all available values) or by entering the first letters of a possible value.

Apart from enabling searching by well-defined chemical structures, the chemical similarity and substructure search enhances the data exploration capabilities of the system (e.g., finding nanoparticles with similar coatings). The data exploration is also supported by REST API calls retrieving data summaries (e.g., number of zeta potential entries) and endpoint prefix queries, allowing for building dashboards and supporting auto-completion fields. Therefore a suitable user interface can be built to allow data search without requiring a priori knowledge of the database content and field names (Figure 14). The search and results retrieval API can be used for many applications, one of which being NanoQSAR modelling. Future extensions, currently under development, include free text search with query expansion based on the eNanomapper ontology and annotated database entries, with an indication of the relevance of the hits. Visual summaries can be integrated in the eNanoMapper web interface, as well as used as widgets in external web sites as demonstrated in the following section.

#### JavaScript visual summaries

To further demonstrate the use of the eNanoMapper API for visualisation we have developed a series of example web pages (HTML) using the JavaScript d3.js library [43]. This library has been used for a wide variety of visualisations (as can be seen on their website), and here used to summarize some of the data in the database. To simplify the interaction with the eNanoMapper API a JavaScript client library, ambit.js, was written to allow asynchronous calls to the web service [44]. However, because the d3.js methods require the data to be provided in a specific JavaScript object, the JSON returned by the API has to be converted to a structure understood by the d3.js code. The sources of the examples presented here are available from the ambit.js project page at http://github.com/enanomapper/ambit.js/. The source code and documentation of the ambit.js library are available at the same location.

**Figure 15:**
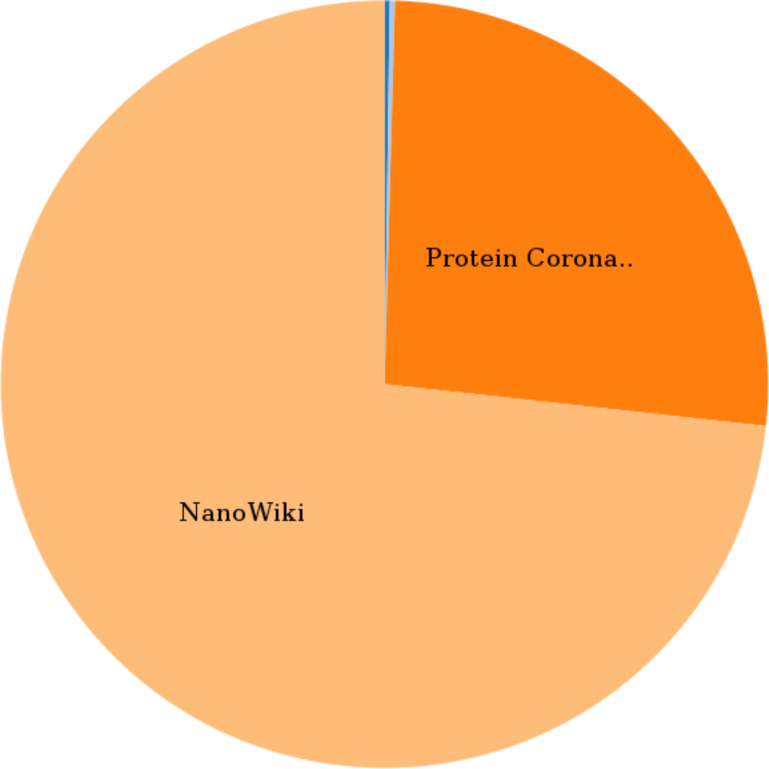
Pie chart created with d3.js and ambit.js in a web page showing that the NanoWiki and Protein Corona datasets contain the most nanomaterials in the database.

**Figure 16:**
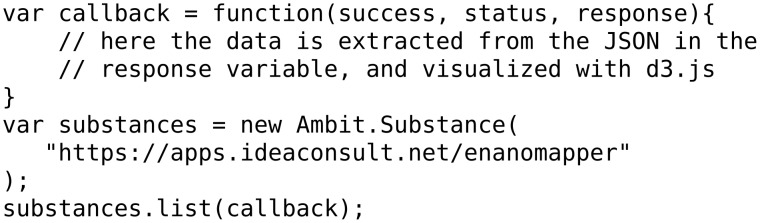
API call in ambit.js code.

The first example shows a summary of the number of materials in the database, sorted by the dataset they originate from (NanoWiki, protein corona, and others), as shown in Figure 15. Here, a single API call was sufficient and the data needed for the pie chart were extracted from the JSON returned by this call. Because of the asynchronous nature of the client–server interaction, a callback function has to be defined. The combination of the callback function (the full implementation is left out for brevity but is available from the ambit.js repository as with Example 2) and the actual API call is done by the ambit.js code given in Figure 16.

**Figure 17:**
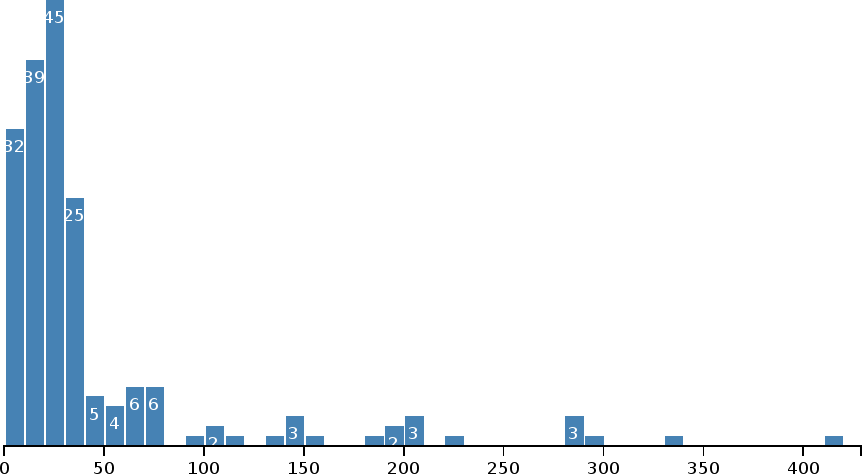
Histogram of nanomaterial sizes created with d3.js and ambit.js.

The second example shows a histogram of nanomaterial sizes (size reported, or average if a size range was given). Because the list of materials does not provide the size information, the callback function of the “Ambit.Substance.list()” call has to make a subsequent call for each material in the list. The example web page keeps track of the number of remaining calls to this second “Ambit.Substance.info()“ API call in a second callback function which also aggregates the material sizes in a global variable. Therefore, the total number of API calls equals the number of materials plus one. When the second callback function notices that there are no further calls to be returned, it calls a plot function that takes the aggregated list of sizes and visualizes it with d3.js, resulting in Figure 17.

**Figure 18:**
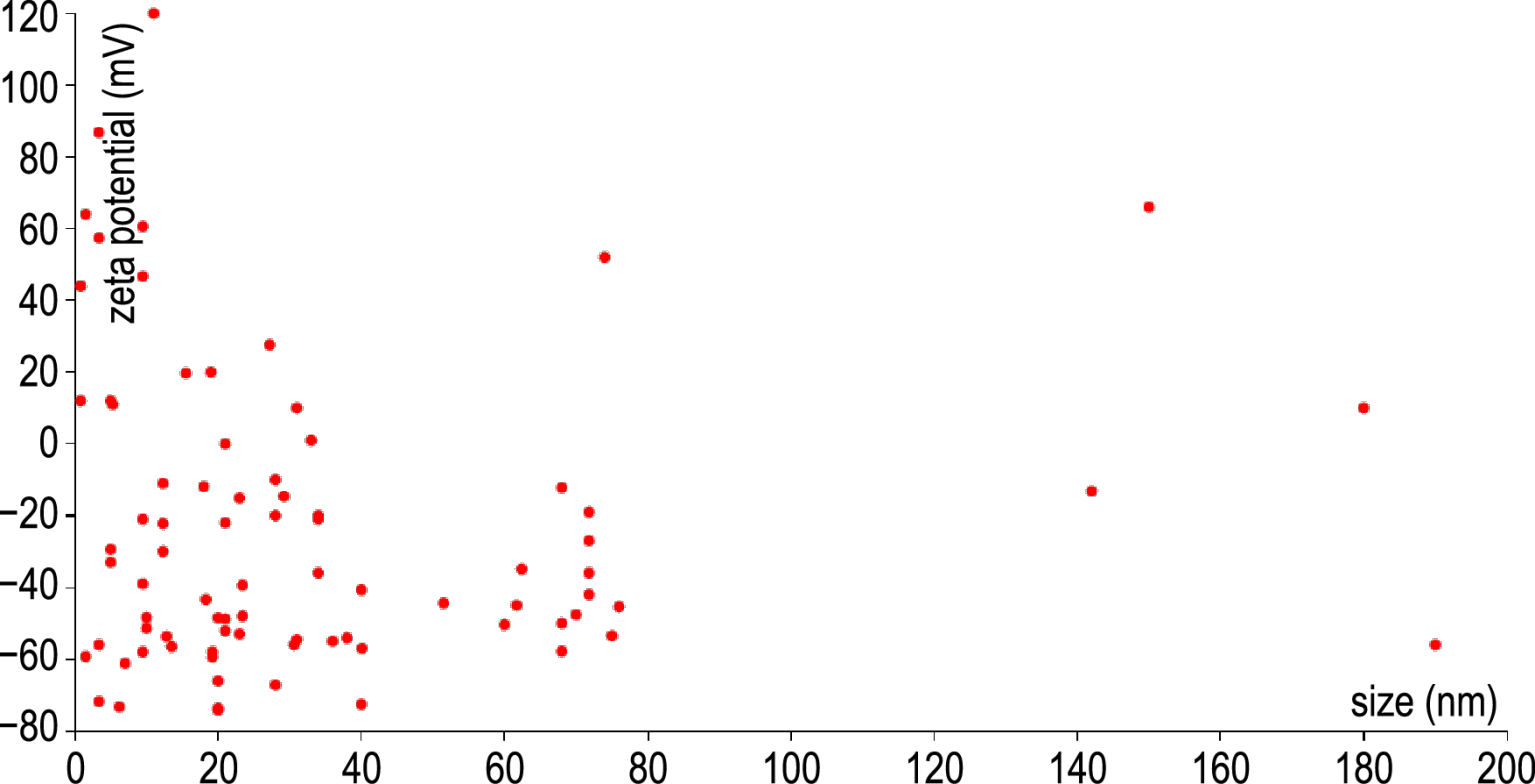
Scatter plot of nanomaterial zeta potentials against the nanomaterial sizes, also created with d3.js and ambit.js.

A variation of the second example shows a scatter plot of the zeta potential values against nanomaterial sizes. Here, the same approach is used and the bits of information are aggregated in a global variable. The results are shown in Figure 18. The red colour of the dots was chosen arbitrarily, but could reflect another feature, possibly the data sources as shown in the first example.

### Modelling

The OpenTox API implementations contain all major statistical and machine learning (ML) algorithms required for the development of regression, classification or clustering models, as well as cheminformatics algorithms, such as structure optimisation and descriptor calculation. A ML algorithm is made available as a web resource and a model is created by sending a HTTP POST to the algorithm URI, with specified dataset URI and modelling parameters, where relevant. The model is again a web resource, and another HTTP POST to the model URI can be used to launch prediction of a specified dataset of chemical structures or materials. However, the OpenTox algorithm and modelling API is centred on chemical structures, and requires clean datasets in a specific form. On the other hand, the eNanoMapper prototype database is explicitly designed to handle all peculiarities of experimental data, including replicates, range and error values. Therefore, a tool, converting the experimental data into a form suitable for modelling algorithms, is required.

**Figure 19:**
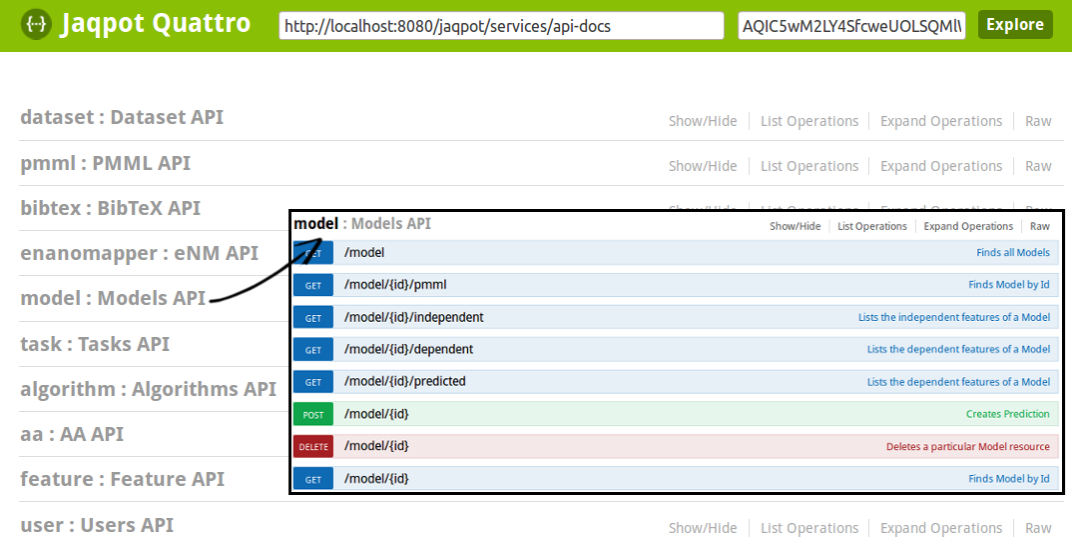
Screenshot of the Jaqpot Quattro modelling web services API, compatible with the eNanoMapper API. A list of REST endpoints is presented to the end user. These correspond to the main entities/resources of eNanoMapper: datasets, models, algorithms, BibTeX entities, asynchronous tasks and more. The user can click on any of these to get a list of the available operations related to each entity. In the inset of this figure we see the list of model-related operations. For more information consult the OpenTox Model API http://opentox.org/dev/apis/api-1.2/Model.

This section describes the approach taken by eNanoMapper, namely the Jaqpot web application, the API documentation of which can be found at http://app.jaqpot.org:8080/jaqpot/swagger, providing one possible solution for this challenge. Jaqpot is a web application that currently supports data preprocessing, statistical, data mining and machine learning algorithms and methods for defining the applicability domain of a predictive model. A screenshot of the Jaqpot web services is presented in Figure 19. Jaqpot provides asynchronous execution of tasks submitted by users, authentication, authorisation and accounting mechanisms powered by OpenAM. It was originally developed during OpenTox [23] and is an open-source project, written in Java and licensed with the GNU GPL v3 licence. Jaqpot Quattro is an extension, developed within eNanoMapper and featuring improved efficiency and additional functionality. Jaqpot Quattro is part of the eNanoMapper framework and communicates with other web services in the framework via the common REST API described above. The source code is publicly available from https://github.com/KinkyDesign/JaqpotQuattro. The main features of Jaqpot Quattro are presented next.

#### Producing datasets from bundles

**Figure 20:**
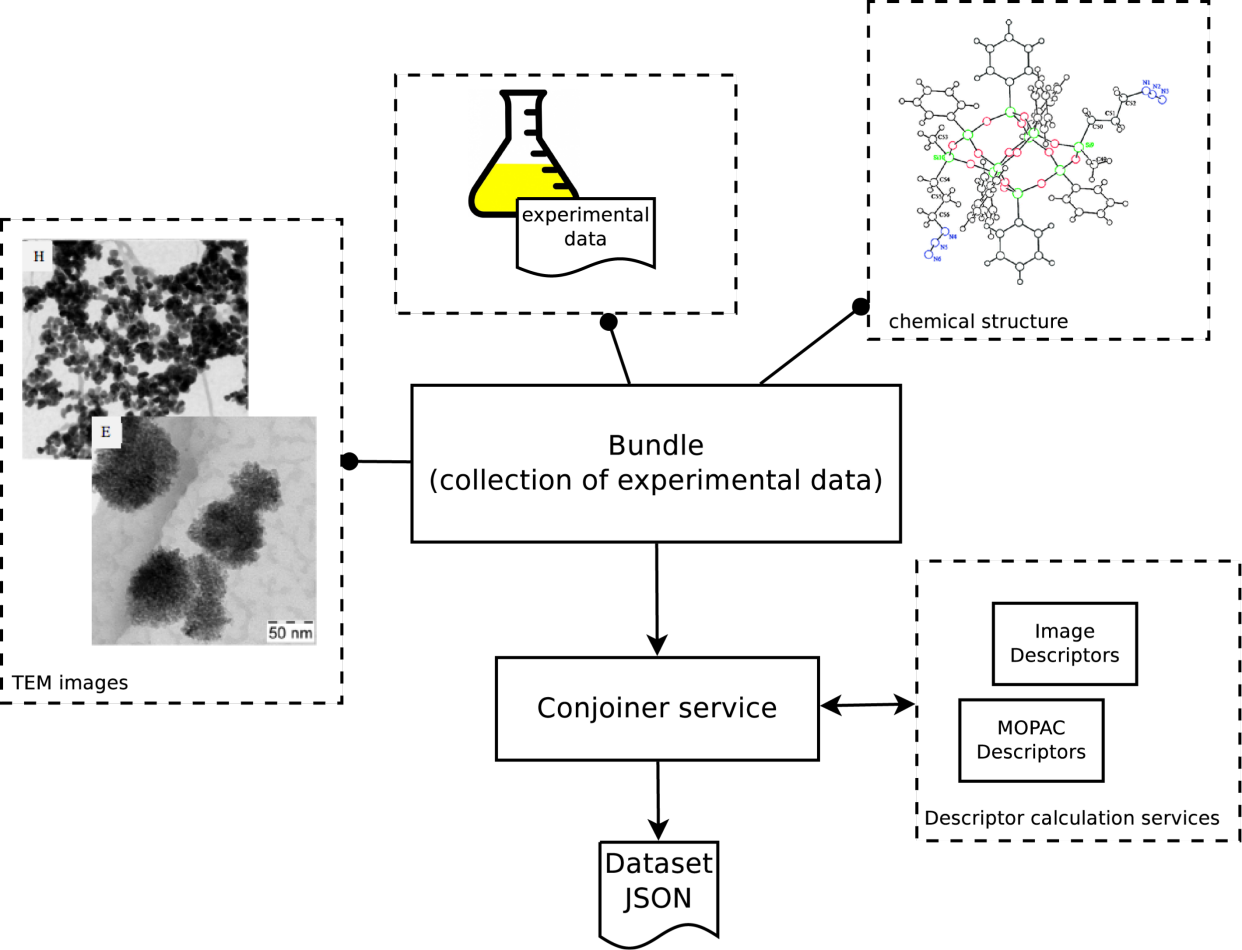
Conjoiner API: modelling-oriented information can be extracted from bundles of experimental data. Data as heterogeneous as chemical structures, raw experimental measurements, spectra and microscopy images can be combined by the conjoiner service to produce a dataset for modelling purposes.

The Jaqpot algorithm services require input data in a standardized format in order to generate a predictive model and raw experimental data cannot be used directly for modelling purposes. The experimental data are, more often than not, heterogeneous by nature and properly structuring these is not a trivial task. To this end, a web service acting as a link between experimental data and data for modelling was introduced, which will be hereafter referred to as the “conjoiner service”. This service performs the task of mapping the experimental data into a modelling-friendly format and producing standardized datasets as specified in the OpenTox API. One can initiate a conjoiner service operation by specifying a bundle URI. A bundle (see Figure 9) is an eNanoMapper resource that acts as an assortment of experimental effects, images and molecular structures, for nanomaterials, and the job of the conjoiner service is to combine all that disparate data into a dataset suitable to be fed to an algorithm service. Concerning experimental data, multiple individual measurements, interval-valued measurements (lower and upper values), or values accompanied by a standard measurement error, may be included for the same endpoint in a bundle, and need to be aggregated into a single value. This is currently done by taking the average value of all experimental measurements having excluded outliers identified by a Dixon’s q-test [45], but different aggregation procedures will be implemented in the future based on more elaborate outlier detection criteria and rejection/aggregation schemata [46–47]. The client will then be able to customise this procedure. The overall procedure is illustrated in Figure 20.

#### Preprocessing

Scaling, normalization and handling of missing values are important preprocessing steps for efficient model training, as most algorithms are sensitive to nonscaled data [48] such as SVM [49]. All these preprocessing steps are offered as options when a client calls a Jaqpot Quattro algorithm service. Furthermore, Jaqpot Quattro makes use of the Predictive Model Markup Language (PMML) file format that allows clients to define a “data dictionary” and a “transformations dictionary”, by providing the URI of a PMML document [50–51]. The data dictionary selects a number of features out of the original dataset that will be provided as inputs to the modelling algorithm, while the transformation dictionary defines mathematical formulae to be applied on the selected features. The predictive model will be then trained using the transformed features as input.

PMML, which has been developed for enabling models to be portable across different computational platforms, is a well-adopted standard in the machine learning and QSAR community. PMML documents are essentially XML documents that contain all necessary information to reproduce a model including the definition of input parameters, targets (predicted properties), preprocessing steps (e.g., scaling, normalization, transformation of inputs), and the main model (e.g., MLR, SVM). The PMML format of the produced NanoQSAR models is also supported by Jaqpot Quattro algorithm services.

**Figure 21:**
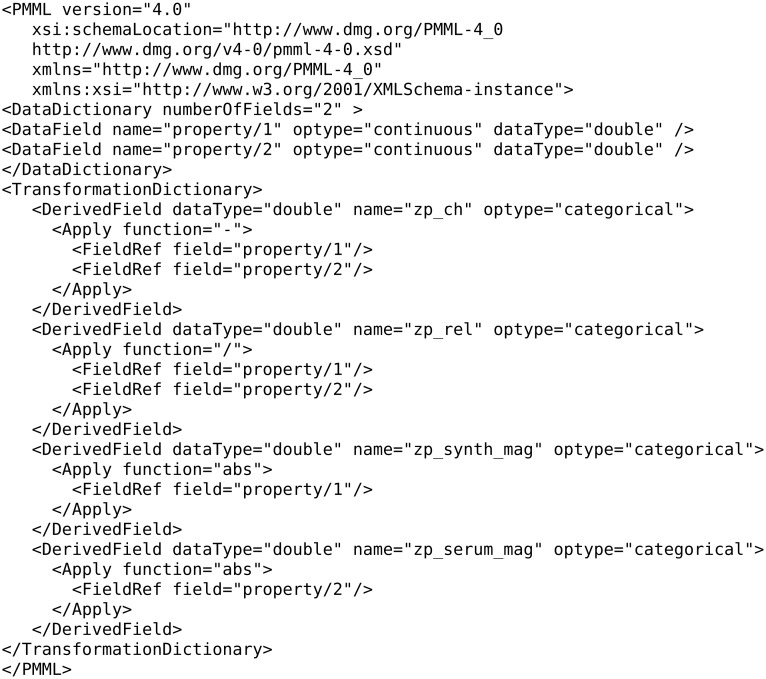
Example of a PMML document.

An example of a PMML document that selects two properties and applies subtraction, division and absolute value operations is given in Figure 21.

Notice that the “DataDictionary” block defines the required input features. The trained model, however, needs to transform these features into the *internal variables* “zp_ch”, “zp_rel”, “zp_synth_mag” and “zp_serum_mag” as specified in the “TransformationDictionary” of the PMML document.

#### API for dynamic algorithm integration

The Jaqpot Protocol of Data Interchange, in short JPDI, is a new feature of the Jaqpot Quattro web services that allows developers of machine learning algorithms to integrate their implementations in the framework. This integration requires little engagement with intricate software development and allows algorithm developers to outsource their implementations and make them available to the nanomaterials design community through the eNanoMapper framework.

**Figure 22:**
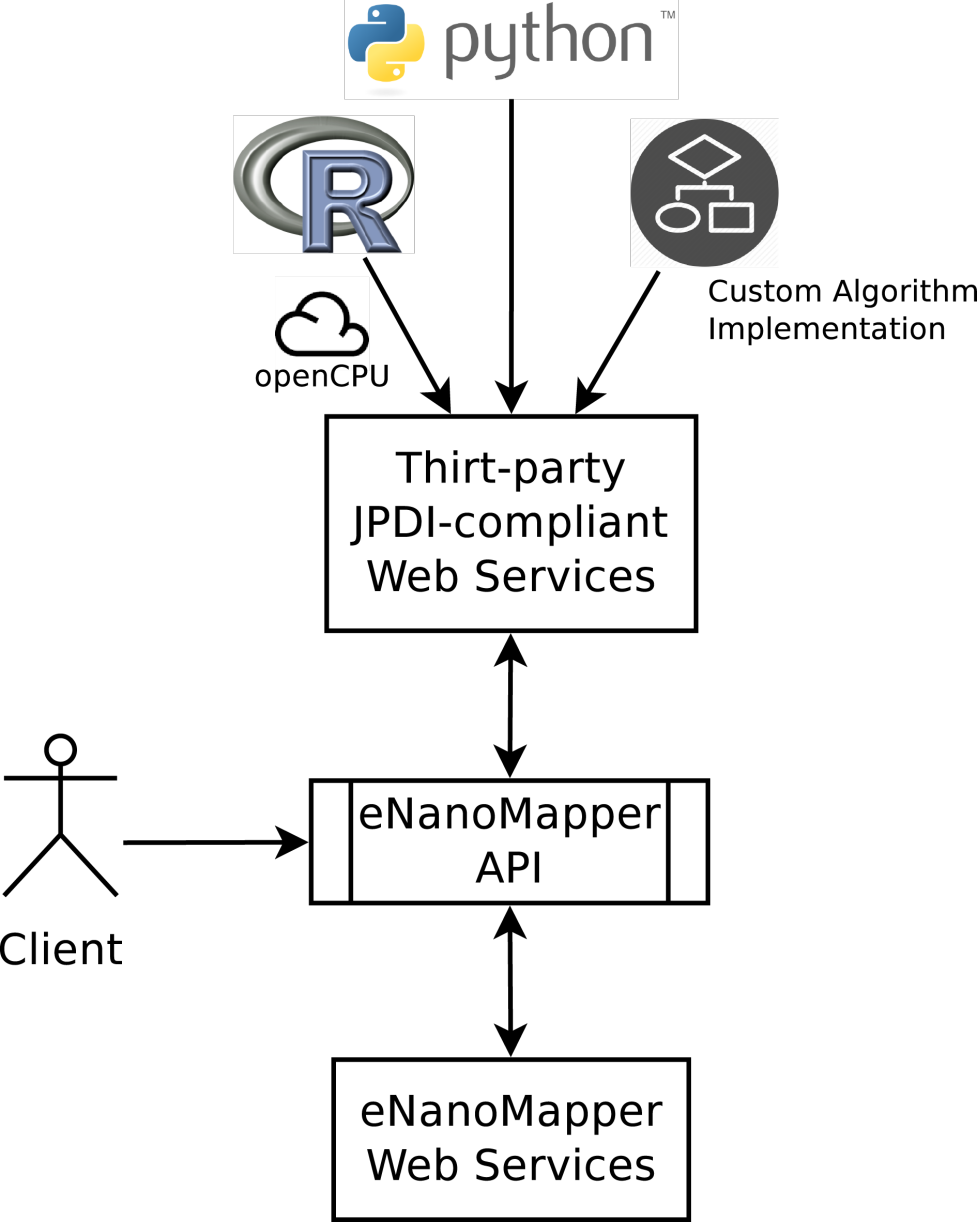
JPDI-compliant web services can be seamlessly incorporated into the eNanoMapper framework. The client communicates with eNanoMapper services through the eNanoMapper API while certain operations such as model training are delegated to JPDI-compliant services.

The communication between eNanoMapper services and third-party JPDI services is carried out by exchanging JSON documents that contain no more information than a modelling service needs to train a predictive model, calculate descriptors, perform a prediction, evaluate the domain of applicability of a model, or perform other tasks. This is well illustrated in Figure 22.

**Figure 23:**
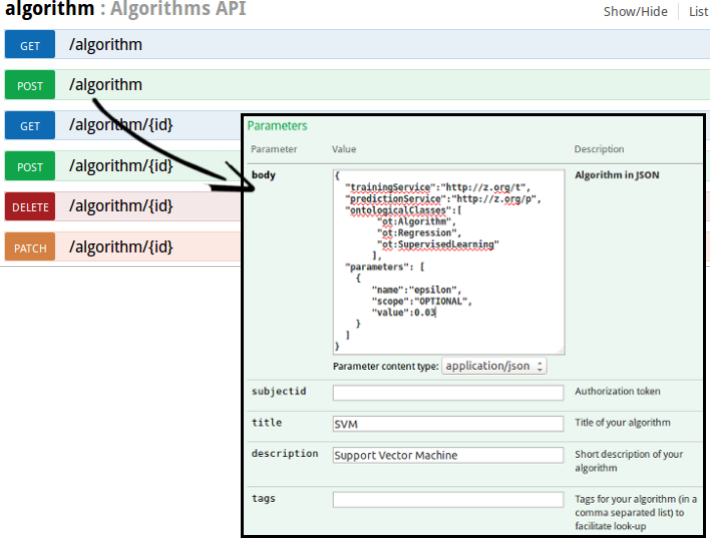
Algorithm API that allows to consume as well as register new algorithms (following the JPDI specification). Clients can use this API to (i) GET a list of all algorithms, (ii) register a new algorithm, (iii) GET the representation of an existing algorithm, (iv) Use an algorithm, (v) Delete an existing algorithm or (vi) use the HTTP method PATCH to modify an algorithm resource.

Once a developer (possibly third-party) has prepared a JPDI-compliant web service, they need to register it to the eNanoMapper framework and specify (i) the name of the algorithm, (ii) metadata for the algorithm, such as a description, tags, copyright notice, bibliographic references and any other metadata supported by the Dublin core ontology (http://dublincore.org/) and/or the OpenTox ontology [52], (iii) the URI of their implementation to be used as an endpoint for training, (iv) the corresponding URI for the prediction web service, (v) an ontological characterization of the algorithm according to the OpenTox Algorithms ontology (e.g., “ot:Regression” or “ot:Classification”, or “ot:Clustering” (http://www.opentox.org/dev/apis/api-1.1/Algorithms), and (vi) a set of tuning parameter definitions, optional or mandatory, that the client may provide during training. The algorithm is then registered by POSTing a JSON document containing all this information to “/algorithm”. Once registered, the algorithm acquires a URI, and is exposed as a web service, that can be consumed. Algorithms can be registered (POST), removed (DELETE) and modified (PATCH) using the Algorithm API presented in Figure 23, which extends the OpenTox Algorithm API (http://opentox.org/dev/apis/api-1.2/Algorithm).

**Figure 24:**
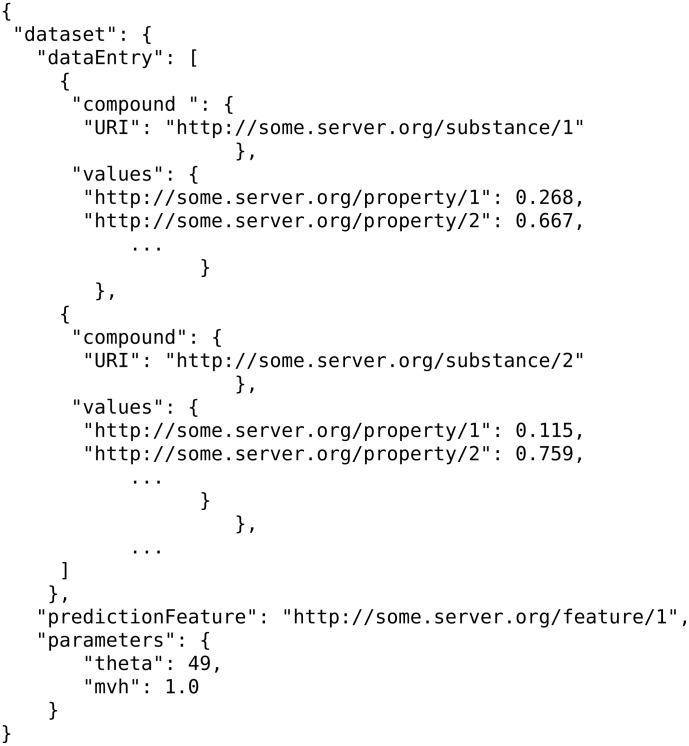
A JPDI request for training.

A JPDI request for training is presented in Figure 24. This request is issued by an algorithm web service of eNanoMapper to a JPDI-compliant web service.

**Figure 25:**
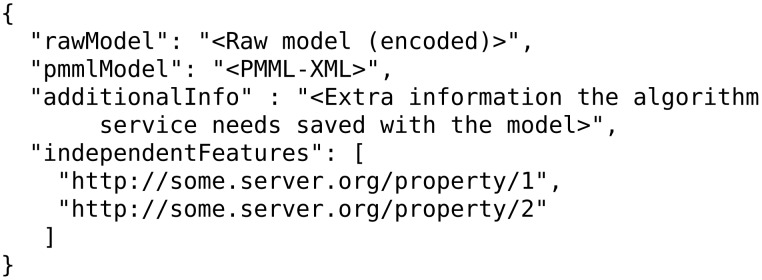
A model returned by JPDI service in JSON format.

Notice the three most important components in a training request, which are the “dataset”, the “prediction feature” and the “tuning parameters” of the algorithm. Once the model is trained, the JPDI service will return it to the caller in JSON format in which the actual model is encoded. Figure 25 gives an example:

Notice that the JPDI web service may select only some of the features of the initial dataset, which are defined in the PMML. Then, the JPDI service requires that a dataset containing these features be posted back to it, i.e., a JPDI service in order to perform predictions requires (i) the model it has previously produced and (ii) a dataset containing values for the features it has selected.

Upon training, the model returned to the caller is stored as-is by the called service and will be returned back to the JPDI-compliant service when the client requests a prediction. This way, as already mentioned, the JPDI service providers do not need to maintain a database while the eNanoMapper services do not need to know how the third-party services perform computations.

**Figure 26:**
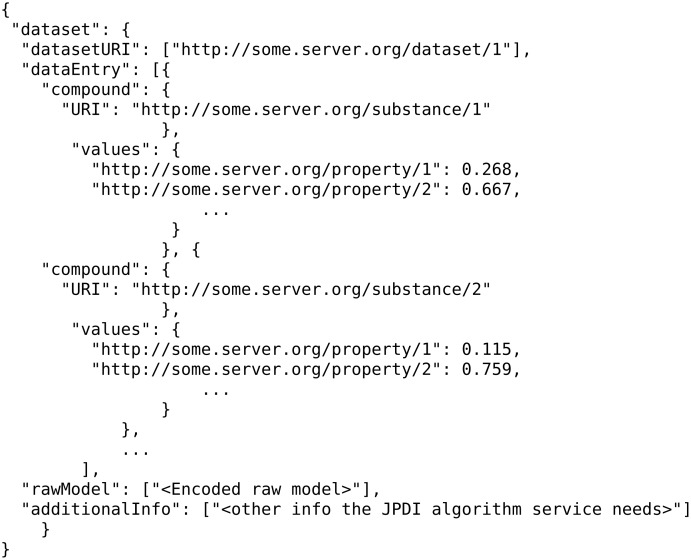
An example of a JSON prediction request.

Likewise, when Jaqpot Quattro needs to consume a JPDI web service to perform predictions, it POSTs to it a JSON document with (i) the input dataset containing substances and (ii) the model that was previously created by the JPDI service. An example of JSON prediction request is shown in Figure 26.

#### Integration with third party services

The JDPI protocol allows one to dynamically and seamlessly incorporate any custom algorithmic implementation into eNanoMapper and without any need for resource management (i.e., the algorithm providers do not need to maintain a database system). The protocol specifies the form of data exchange between eNanoMapper services and third party algorithm web service implementations. The eNanoMapper framework already provides wrappers for WEKA [53] and the R language [54]. Integration with R is made possible through the OpenCPU (https://www.opencpu.org/) system, which defines a HTTP API for embedded scientific computing based on R although this approach could easily be generalized to other computational back ends [55]. OpenCPU acts as a wrapper to R that is readily able to expose R functions as RESTful HTTP resources. The OpenCPU server takes advantage of multi-processing in the Apache2 web server to handle concurrency. This implementation uses forks of the R process to serve concurrent requests immediately with little performance overhead. By doing so it enables access to those functions on simple HTTP calls converting R from a stand-alone application to a web service. R (http://www.r-project.org/) has become the most popular language for computational statistics, visualization and data science, in both academia and industry [56]. One of the most important benefits for R users is cost-free, easy access to the frontline of methods in predictive modelling and statistics that are produced and are under continuous review from leading data science researchers [57]. In Bioinformatics, the Bioconductor R branch (http://www.bioconductor.org/), provides open source tools for high-throughput omic data analysis. Bioconductor users enjoy access to a wide array of statistical and graphical methods for genomic data analysis and makes it much easier to incorporate biological metadata in genomic data analysis, e.g., PubMed literature data (http://www.ncbi.nlm.nih.gov/pubmed), annotation data extracted from Entrez genes, etc. This is one of its important features, since users can easily gather all the relevant biological information and analyse their integrated findings or validate their results. We are planning on integration with other software packages, developed in Matlab (or Octave) and Python. Python is gaining considerable momentum for machine learning applications as various packages facilitate the analysis of data, development and validation of models, conduction of various statistical analyses and other tasks. Scikit-learn (http://scikit-learn.org/stable/), pyBrain (http://pybrain.org), and mlpy (http://mlpy.sourceforge.net/) are a few of the numerous machine learning packages for Python.

#### Algorithm Implementations

Currently, Jaqpot Quattro contains the following API-compliant algorithm services: two implementations of multiple linear regressions (MLR) (using R and Weka [53] functionalities), and implementations of the partial least squares (PLS) algorithm (based on Weka), the support vector machine method (using the LIBSVM library [58]) and the sub-clustering algorithm developed in-house for Radial Basis Function Neural Networks [59]. As an example, the R implementation of the MLR regression algorithm was applied on the corona dataset to generate a linear NanoQSAR model that relates net cell association of gold nanoparticles (the logarithm base 2 transformed values) to zeta potential after synthesis, zeta potential after serum exposure, and a number of transformation defined in the PMML file found at http://app.jaqpot.org:8080/jaqpot/services/pmml/corona-standard-transformations. The produced model, trained with the algorithm with ID “ocpu-lm” (located at http://app.jaqpot.org:8080/jaqpot/services/ocpu-lm) can be found under the following address: http://app.jaqpot.org:8080/jaqpot/services/model/corona-model. OCPU-LM is implemented in R (using OpenCPU) and exposed via the JPDI API as explained in the previous section. To access these resources the client needs to provide an authentication token as specified by the access control API. Alternatively, the end user can easily access it via the Jaqpot Swagger interface (http://app.jaqpot.org:8080/jaqpot/swagger) using an authorization token produced automatically.

**Figure 27:**
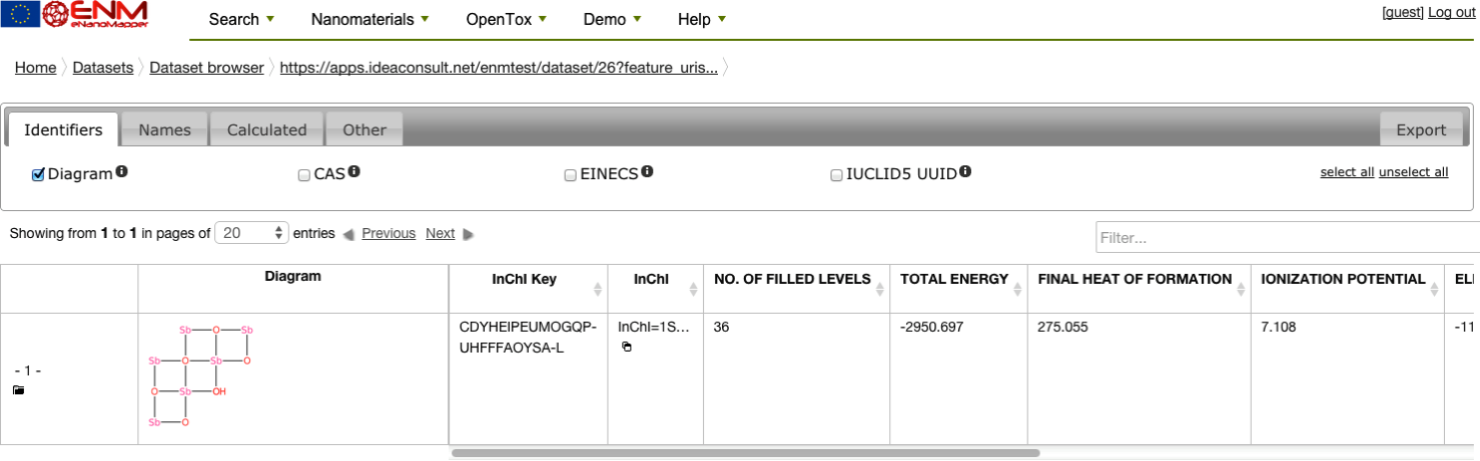
Screenshot of the descriptors calculated with quantum mechanics MOPAC web service.

Apart from experimental descriptors available through the database, datasets used for modelling may contain theoretical descriptors, which are calculated using services that were originally developed during the OpenTox project, but are now being updated and extended, such as CDK [60] and MOPAC [61]. The eNanoMapper MOPAC implementation (available at: https://apps.ideaconsult.net/enanomapper/algorithm/ambit2.mopac.MopacOriginalStructure) was used to calculate quantum-mechanical descriptors for metal oxides, including HOMO (highest occupied molecular orbital), LUMO (lowest unoccupied molecular orbital), band gap and ionization potential. Figure 27 shows the results for Sb_2_O_3_ (available at http://enanomapper.github.io/bjnano7250433 ; login as guest is required for access). Calculations are available in various formats, including CSV, JSON, CML and SDF.

The leverage method for defining the “applicability domain” (AD) of NanoQSAR models has also been implemented and offered as a service. According to the OECD definition, the “applicability domain of a (Q)SAR model is the response and chemical structure space in which the model makes predictions with a given reliability” [62–63]. Defining the chemical structure space for nanomaterials is not trivial, hence the descriptor-based approach is adopted. The AD is created by applying a POST at an instance of the AD web service. Then, the predictive model can be linked to the AD model in such a way that predictions are accompanied by an indicator that informs us whether the query compound is in or out of the AD of the model.

### Integration of modelling services in the framework

Dataset resources from any dataset service may be used by any modelling service which in turn will store the produced dataset of prediction on any dataset service. The eNanoMapper web services design assumes a distributed architecture in which data are not required to be stored or even indexed by a common system. Among services that implement the API, input data can come from any dataset service, be used by any modelling service, which in turn will submit the produced dataset with prediction results to any dataset service for storage. Linked-data principles are combined here with a REST-based design to enable this distribution of resources.

## Discussion

The API with resources supporting substances, protocols and measurements is in line with recent publications in the domain and is able to support a variety of tests and endpoints, recommended by the OECD WPMN. The annotation with ontology entries is an ongoing collaboration between the eNanoMapper database and ontology teams and the EU NanoSafety Cluster. Data heterogeneity is a pervasive challenge within the nanosafety domain, with the complexity of the nanomaterials and their biological interactions being measured via multiple different types of assays and endpoints across a wide range of experimental technologies. While our prototype database and ontology already illustrate a range of these different measurements, the list of possible endpoints and characterisation properties is growing all the time as the science evolves, and our objective is ultimately to represent all relevant properties and endpoints in our ontology, which is currently growing through community feedback and as it is being used for annotations. Given the heterogeneity of the data being represented, a challenge of inconsistency may also emerge. Our platform is inspired by the OECD recommendations to define a minimum set of information that needs to be included as metadata in the case of each experiment type. Through templates, the fields that are required for different protocols can be customised.

The demonstration data provided by partners illustrates the capability of the API and the implementation to handle diverse information. It has been used for NanoQSAR modelling. Research is ongoing to extend the OpenTox algorithm and modelling APIs for nanomaterials, allowing these new models to be exposed with unique URIs suitable for reuse. The REST API with JSON serialisation is the current state of the art in web system development and data integration and enables building graphical summaries of the data, JavaScript widgets, custom user interfaces and programmatic interaction. The next steps include provision of RDF serialisation of the resources, support for multiple data formats on import and export, support for multiple search interfaces (including ones based on semantic technologies), and improvements of the data model, API and the implementation, based on the feedback and close collaboration with all eNanoMapper partners and EU NanoSafety Cluster working groups.

The eNanoMapper database discussed here is a design architecture that allows, in a first stage, for the import of experimental data and calculated descriptors by those who have measured or calculated them respectively, and in a second stage the use of data from the database for propagation or modelling. The eNanoMapper team from the beginning of the project paid attention to designing a system that would be strict in enforcing traceability of data, and in recording the details in its representation of nanomaterials and the specifics of how the data were generated (experimental conditions, methods). Users of the platform prototype feed it with data they have curated and know to be accurate. Any problematic uploads can be traced back to their source. In future work, metrics on the data such as the compliance level suggested by the Nanomaterial Registry (https://www.nanomaterialregistry.org/about/HowIsComplianceCalculated.aspx) could be introduced in order to progress the nanomaterial safety community towards a holistic approach to data quality that may be triggered from data storage, but this also needs to go back to the data origins, i.e., the specifics of experiments/measurements/calculations. Besides being accessible online at data.enanomapper.net, the system presented is an open source solution, which can be downloaded, installed and hosted by individual researchers or labs, and as such presents an open distributed platform for NM data management, rather than being restricted to use as a single database instance.

### Data format conversions

Formatting experimental data as ISA-Tab files manually is very cumbersome and time consuming, even if using “semantically aware” tools, such as ISAcreator (https://github.com/ISA-tools/ISAcreator). Formatting data as ISA-Tab-Nano is even more challenging, as there is no publicly available validator of ISA-Tab-Nano, and the available examples at https://wiki.nci.nih.gov/display/ICR/ISA-TAB-Nano are more useful to convey the idea of the format, rather than to be considered the ultimate specification-compliant instances. Furthermore, while ISA-Tab validation relies heavily on XML assay templates, specifying the fields required by experiments with a defined endpoint and technology, the ISA-Tab-Nano wiki does not provide such templates, which makes it impossible to use existing ISA-Tab tools to generate ISA-Tab-Nano compliant files, even if ignoring the ENM-specific material files. Last but not least, the ISA-Tab specification only defines the metadata format and does not impose any restrictions on the actual data files. We consider two parallel roads towards improvement of the status quo. First, enabling ISA-Tab-Nano support by the core ISA-Tab tools (ISAcreator), and second, an automatic generation of ISA-Tab archives, given the ubiquitous and convenient Excel templates as input. We have initiated work towards the first goal by extending a fork of the ISA-Tab core code to enable parsing of ISA-Tab-Nano files (https://github.com/enanomapper/ISAvalidator-ISAconverter-BIImanager). As this code is part of the ISAcreator application, it would potentially allow for loading and validating of ISA-Tab-Nano files through the core ISA-Tab tools. While ISA-Tab is designed to ensure that all experimental details are retained, the chemical compound or ENM is hidden in a step of the experimental graph, and such a data model is usually less convenient for preparing and querying the data and applying subsequent predictive modelling. Building on previous experience and taking into account the observation that the majority of EU NanoSafety Cluster projects prefer to prepare their experimental data using custom spreadsheet templates, the eNanoMapper team took an alternative, but pragmatic, approach by implementing support for a large set of custom spreadsheet templates for data preparation. We developed the configurable Excel parser described in the “Data import” section above. Being able to parse diverse spreadsheets, as well as other input formats (such as OHT) into the same internal data model and export the data from this data model into different formats allows us to provide format converters, in the same fashion as OpenBabel [64] (http://openbabel.org/) interconverts between chemical formats. Extending the tools to include ontology annotations and to be able to write the internal data model into ISA-Tab files will not only accomplish the second goal of automatically generating the files, but will also enable exporting query results from the database in a desired format.

### Modelling

We are now developing a new R package that automates the creation of the best possible NanoQSAR regression model (validated using cross validation and external testing), by searching over many different regression algorithms and tuning the parameters for each algorithm. The suggested workflow automates the development of a reliable and well-validated NanoQSAR model or set of models by a simple call to an R function. The R package will be integrated within the eNanoMapper system using the JDPI and OpenCPU functionalities, described before.

Transmission electron microscopy (TEM) is a valuable technique for the characterization of nanomaterials. TEM image analysis yields number-based results, allows the extraction of size and shape-related attributes and characterization of surface topologies, and provides distinctions between the characterizations of primary particles and of aggregates/agglomerates. Based on TEM images, Gajewicz et. al. have proposed a set of image-derived descriptors for characterizing nanomaterials, such as volume, area, porosity and circularity [65]. These descriptors will be included in the set of descriptors to be computed by an image analysis tool that is under development in the context of the eNanoMapper project based on the standard and well accepted Fiji/ImageJ [66] open-source software, which was selected after an assessment of the most relevant software tools that are available and in use by the scientific community.

Integration of the facilities provided by R will allow for easy access to a wealth of additional algorithms and methods focusing on the analysis of omics data and utilization of useful information included in public ontologies such as the Kyoto Encyclopedia of Genes and Genomes (KEGG) [67]. Recent studies suggest that integrating multi-omics additional genomic knowledge can greatly assist towards fully understanding various phenotypes [68], as opposed to the conclusions drawn by focusing on only a single level of genomic data. Along these lines, we are working on an integration clustering analysis of the proteomics data included in protein corona datasets also incorporating information from the underlying relations in the data using the Gene Ontology [69]. For example, a hierarchical clustering algorithm is applied to NP proteomics data to build a hierarchy of protein clusters and compare them to those established by Gene Ontology; similarities between the two should reinforce any toxicology related outcome.

### Technology

The REST API has become the most commonly used approach for web application development. Because of its simplicity and performance scalability it has replaced solutions such as the simple object access protocol (SOAP). The OpenTox project was in 2008 one of the first to define and implement a REST API in the cheminformatics and QSAR domains [14,23,70], but nowadays all the major chemical (and some material) databases provide access via REST. This applies to both data as well as computational functionality, including wrappers for popular software as R, science-as-a-service platforms, and high-performance computing, because the demand for interfacing via web services increases. REST is defined as a software architecture style designated for network-based applications, as the outcome of a thorough analysis of network architectures [71]. It is compliant with the successful architectural principles behind the World Wide Web that characterizes RESTful applications. Specifically, the principles were selected to ensure the distributed system will feature a set of particular properties: simplicity, scalability, performance, modifiability, visibility of communication, portability, reliability, and resistance to failure. The granularity of the REST resources is not fixed, but can be designed to fit particular application needs. A set of resources is a resource itself, hence there are no efficiency limitations on the retrieval of large amounts of data. A potential challenge when processing large amounts of data is the output in textual format, however a resource representation can be compressed, and in principle the JSON output used is much more terse than other formats (e.g., RDF). REST allows for the provision of representations in multiple formats, hence formats suitable for representing sparse data can be utilized. A known limitation is that a REST API specifies how questions can be asked and therefore restricts the users in what they can ask, compared to being able to access the data directly via, e.g., SPARQL (SPARQL protocol and RDF query language) or SQL (structured query language). While eNanoMapper plans to enable SPARQL queries for NM data, this approach has its own drawbacks which often motivate the hiding of SPARQL behind a REST API. Despite the overwhelming use of REST with HTTP protocol and HTTP URIs, originally REST was a protocol-independent architecture and could be used outside of the HTTP context, which, in principle, allows for the adoption of binary protocols for effectiveness (such as Google protocol buffers, Apache Thrift, etc.). However binary protocols are much harder to use. We do not expect a solution other than HTTP to be required in the lifetime of the eNanoMapper project. Finally, it deliberately adopts the choice of a distributed database system, which follows the same idea as the World Wide Web, and is in accordance with the REST architecture of an ecosystem of distributed entities that interact and are made available independently from each other.

## Conclusion

The eNanoMapper database builds on previous experience from the OpenTox and ToxBank projects in supporting diverse data through flexible data storage, semantic web technologies, open source components and web services. A number of opportunities and challenges exist in nanomaterials representation and integration of ENM information, originating from diverse systems. We adopted the concept of substances, allowing a more elaborate representation of ENMs, overcoming limitations of existing compound-based databases and integration solutions. We describe how an approach of adopting an ontology-supported data model, covering substances and measurements, provides a common ground for integration. The data sources supported include diverse formats (ISA-Tab, OECD harmonized templates, custom spreadsheet templates), as well as other formats via custom import scripts. Besides retaining the data provenance, the focus on measurements provides insights into how to reuse chemical structure database tools for nanomaterials characterization and safety.

The database is still under development within the eNanoMapper project. Future work includes support for high-throughput screening (HTS) data, further annotation with ontologies, and support for data from aforementioned third-party databases, such as PubChem and ArrayExpress. HTS and high-content analysis data are currently being generated in several of the projects within the EU NanoSafety Cluster, including the eNanoMapper partners. As these datasets become available, they will be able to serve in generating use cases for further development, refinement and proof-of-concept of the current state of the eNanoMapper database and ontology framework.

Nanomaterials synthesis until the final product stage may potentially involve several analyses, where go, no-go decisions are made from evaluating safety and other aspects of the materials. Ultimately, we envision that the eNanoMapper infrastructure should be directly applicable to such a safe-by-design principle, directly coupling the material and product development stages with safety analysis. The current prototype provides us with the means of comparing new nanomaterials to an expanding collection of reference data.

## Supporting Information

File 1 OECD WPMN recommended endpoints and their potential correspondence to UDS and ISA-Tab-Nano concepts.
