# Supplementary material for: The eNanoMapper database for nanomaterial safety information
Source: Beilstein J Nanotechnol. 2015 Jul 27;6:1609–34. doi: 10.3762/bjnano.6.165 (PMC4578352; doi:10.3762/bjnano.6.165)
Supplement: File 1 — OECD WPMN recommended endpoints and their potential correspondence to UDS and ISA-Tab-Nano concepts. [file Beilstein_J_Nanotechnol-06-1609-s001.pdf]

Supporting Information

for

The eNanoMapper database for nanomaterial safety information

Nina Jeliazkova*[1], Charalampos Chomenidis[2], Philip Doganis[2], Bengt Fadeel[3], Roland Grafström[3], Barry Hardy[4], Janna Hastings[5], Markus Hegi[4], Vedrin Jeliazkov[1], Nikolay Kochev[1,6], Pekka Kohonen[3], Cristian R. Munteanu[7,8], Haralambos Sarimveis[2], Bart Smeets[7], Pantelis Sopasakis[2,9], Georgia Tsiliki[2], David Vorgrimmler[10] and Egon Willighagen[7]

Address: [1]Ideaconsult Ltd., Sofia, Bulgaria; [2]National Technical University of Athens, School of Chemical Engineering, Athens, Greece; [3]Karolinska Institutet, Stockholm, Sweden; [4]Douglas Connect GmbH, Zeiningen, Switzerland; [5]European Molecular Biology Laboratory – European Bioinformatics Institute (EMBL-EBI), Hinxton, United Kingdom; [6]University of Plovdiv, Department of Analytical Chemistry and Computer Chemistry, Plovdiv, Bulgaria; [7]Department of Bioinformatics, NU-TRIM, Maastricht University, Maastricht, Netherlands; [8]Computer Science Faculty, University of A Coruna, A Coruña, Spain; [9]IMT Institute for Advanced Studies Lucca, Lucca, Italy and [10]in silico toxicology Gmbh (IST), Basel, Switzerland

Email: Nina Jeliazkova* - jeliazkova.nina@gmail.com

* Corresponding author

OECD WPMN recommended endpoints

#	Endpoints agreed by the OECD WPMN	OECD HT (XML schema)	CODATA UDS	ISA-TAB-Nano
**Nanomaterial Information / Identification**				
1	Nano material name	SUBSTANCE	Information Category/ General Identifiers	Material file
2	CAS number	SUBSTANCE	Information Category/ Characterisation/Chemical composition	Material file
3	Structural formula/molecular structure	SUBSTANCE	Information Category/ Characterisation/Chemical composition	Material file
4	Composition of NM being tested (incl. degree of purity, known impurities or additives)	SUBSTANCE	Information Category/ Characterisation/Chemical composition; Information Category/ Characterisation/Physical structure	Material file
5	Basic Morphology	GI_GENERAL_INFORM	Information Category/ Characterisation/Physical structure	Material file
6	Description of surface chemistry (e.g. coating or modification)	SUBSTANCE SURFACE_CHEMISTRY	Information Category/ Characterisation/Surface description	Material file (nominal) Assay file (measured)
7	Major commercial uses	PRODUCT_TYPE_USE, DIRECTIONS_FOR_USE		
8	Known catalytic activity	CATALYTIC_ACTIVITY	Information Category/ Characterisation/Intensive properties	Assay file
9	Method of production (e.g. precipitation, gas phase)	SUBSTANCE	Information Category/ Production, Specification	Study file (protocol)
**Physical-chemical Properties and Material Characterization**				
10	Agglomeration / aggregation	AGGLOMERATION_AGGREGATION	Information Category/ Characterisation/Physical structure	Assay file, Data file
11	Water solubility	PC_WATER_SOL	Information Category/ Characterisation/Interaction	Assay file, Data file
12	Crystalline phase	CRYSTALLINE_PHASE	Information Category/ Characterisation/ Crystallographic structure	Assay file, Data file
13	Dustiness	DUSTINESS		Assay file, Data file

14	Crystallite size	CRYSTALLITE_AND_GRAIN_SIZE	Information Category/ Characterisation/ Crystallographic structure	Assay file, Data file
15	Representative TEM picture(s)	ATTACHMENTDOCUMENT		Assay file, Data file
15	Particle size distribution	PC_GRANULOMETRY	Information Category/ Characterisation/ Size	Assay file, Data file
17	Specific surface area	SPECIFIC_SURFACE_AREA	Information Category/ Characterisation/ Surface description	Assay file, Data file
18	Zeta potential (surface charge)	ZETA_POTENTIAL_SECTION	Information Category/ Characterisation/Interaction	Assay file, Data file
19	Surface chemistry (where appropriate)	SURFACE_CHEMISTRY	Information Category/ Characterisation/Surface description	Assay file, Data file
20	Photo-catalytic activity	PHOTOCATALYTIC_ACTIVITY	Information Category/ Characterisation/Interaction	Assay file, Data file
21	Pour density	POUR_DENSITY	Information Category/ Characterisation/Intensive properties	Assay file, Data file
22	Porosity	POROSITY	Information Category/ Characterisation/Intensive properties	Assay file, Data file
23	Octanol-water partition coefficient, where relevant	PC_PARTITION	Information Category/ Characterisation/Intensive properties	Assay file, Data file
24	Redox potential		Information Category/ Characterisation/Interactions	Assay file, Data file
25	Radical formation	RADICAL_FORMATION_POTENTIAL	Information Category/ Characterisation/Interactions	Assay file, Data file
26	Other relevant information (where available)	PC_OTHER		Assay file, Data file
**Environmental Fate**				
27	Dispersion stability in water			Assay file, Data file
	**Biotic degradability**			
28	- Ready biodegradability	TO_BIODEG_WATER_SCREEN	Information Category/ Characterisation/Interactions	Assay file, Data file
29	- Simulation testing on ultimate degradation in surface water	TO_BIODEG_WATER_SIM	Information Category/ Characterisation/Interactions	Assay file, Data file
30	- Soil simulation testing		Information Category/	Assay file, Data

			Characterisation/Interactions	file
31	Sediment simulation testing		Information Category/ Characterisation/Interactions	Assay file, Data file
32	- Sewage treatment simulation testing		Information Category/ Characterisation/Interactions	Assay file, Data file
33	Identification of degradation product(s)	EN_MAIN_DEGRADATION	Information Category/ Characterisation/Interactions	Assay file, Data file
34	Further testing of degradation product(s) as required		Information Category/ Characterisation/Interactions	Assay file, Data file
	**Abiotic degradability and fate**			
35	- Hydrolysis, for surface modified nanomaterials	TO_HYDROLYSIS	Information Category/ Characterisation/Interactions	Assay file, Data file
36	Adsorption- desorption	EN_ADSORPTION	Information Category/ Characterisation/Interactions	Assay file, Data file
37	Adsorption to soil or sediment	EN_STABILITY_IN_SOIL	Information Category/ Characterisation/Interactions	Assay file, Data file
38	Bioaccumulation potential	EN_BIOACCUMULATION	Information Category/ Characterisation/Interactions	Assay file, Data file
39	Bioaccumulation in sediment	EN_BIOACCU_TERR	Information Category/ Characterisation/Interactions	Assay file, Data file
**Environmental Toxicology**				
40	Effects on pelagic species (short/ long term)	EC_FISHTOX, EC_CHRONFISHTOX, EC_DAPHNIATOX, EC_CHRONDAPHNIATOX, EC_ALGAETOX	Information Category/ Characterisation/Interactions	Assay file, Data file
41	Effects on sediment species (short/ long term)	EC_SEDIMENTDWELLINGTOX	Information Category/ Characterisation/Interactions	Assay file, Data file
42	Effects on soil species (short/ long term)	EC_SOILDWELLINGTOX	Information Category/ Characterisation/Interactions	Assay file, Data file
43	Effect on terrestrial species	EC_PLANTTOX, EC_HONEYBEESTOX	Information Category/ Characterisation/Interactions	Assay file, Data file
44	Effect on micro-organisms	EC_SOIL_MICRO_TOX, EC_BACTOX	Information Category/ Characterisation/Interac	Assay file, Data file

			tions	
45	Other relevant information		Information Category/ Characterisation/Interactions	Assay file, Data file
**Mammalian Toxicology**				
46	Pharmacokinetics (ADME)		Information Category/ Characterisation/Interactions	Assay file, Data file
47	Acute Toxicity	TO_ACUTE_ORAL, TO_ACUTE_INHAL, TO_ACUTE_DERMAL	Information Category/ Characterisation/Interactions	Assay file, Data file
48	Repeated dose toxicity	TO_REPEATED_ORAL, TO_REPEATED_INHAL TO_REPEATED_DERMAL	Information Category/ Characterisation/Interactions	Assay file, Data file
49	Chronic toxicity	TO_CARCINOGENICITY, TO_SENSITIZATION	Information Category/ Characterisation/Interactions	Assay file, Data file
50	Reproductive toxicity	TO_REPRODUCTION	Information Category/ Characterisation/Interactions	Assay file, Data file
51	Developmental toxicity	TO_DEVELOPMENTAL	Information Category/ Characterisation/Interactions	Assay file, Data file
52	Genetic toxicity	TO_GENETIC_IN_VITRO, TO_GENETIC_IN_VIVO	Information Category/ Characterisation/Interactions	Assay file, Data file
53	Experience with human exposure	TO_EXPOSURE_OTHER	Information Category/ Characterisation/Interactions	Assay file, Data file
54	Other relevant test data	TO_OTHER	Information Category/ Characterisation/Interactions	Assay file, Data file
**Material Safety**				
55	Flammability	PC_AUTO_FLAMM, PC_FLAMM	Information Category/ Characterisation/Interactions	Assay file, Data file
56	Explosivity		Information Category/ Characterisation/Interactions	Assay file, Data file
57	Incompatibility		Information Category/ Characterisation/Interactions	Assay file, Data file
